# Phenocopy – A Strategy to Qualify Chemical Compounds during Hit-to-Lead and/or Lead Optimization

**DOI:** 10.1371/journal.pone.0014272

**Published:** 2010-12-10

**Authors:** Patrick Baum, Ramona Schmid, Carina Ittrich, Werner Rust, Katrin Fundel-Clemens, Susanne Siewert, Martin Baur, Lisa Mara, Lore Gruenbaum, Armin Heckel, Roland Eils, Roland E. Kontermann, Gerald J. Roth, Florian Gantner, Andreas Schnapp, John E. Park, Andreas Weith, Karsten Quast, Detlev Mennerich

**Affiliations:** 1 Boehringer Ingelheim Pharma GmbH & Co. KG, Biberach an der Riss, Germany; 2 Institute of Cell Biology and Immunology, University of Stuttgart, Stuttgart, Germany; 3 Boehringer Ingelheim Pharmaceuticals Inc., Ridgefield, Connecticut, United States of America; 4 Institute of Pharmacy and Molecular Biotechnology/BIOQUANT, University of Heidelberg, Heidelberg, Germany; University of Birmingham, United Kingdom

## Abstract

A phenocopy is defined as an environmentally induced phenotype of one individual which is identical to the genotype-determined phenotype of another individual. The phenocopy phenomenon has been translated to the drug discovery process as phenotypes produced by the treatment of biological systems with new chemical entities (NCE) may resemble environmentally induced phenotypic modifications. Various new chemical entities exerting inhibition of the kinase activity of Transforming Growth Factor β Receptor I (TGF-βR1) were qualified by high-throughput RNA expression profiling. This chemical genomics approach resulted in a precise time-dependent insight to the TGF-β biology and allowed furthermore a comprehensive analysis of each NCE's off-target effects. The evaluation of off-target effects by the phenocopy approach allows a more accurate and integrated view on optimized compounds, supplementing classical biological evaluation parameters such as potency and selectivity. It has therefore the potential to become a novel method for ranking compounds during various drug discovery phases.

## Introduction

A phenocopy is defined as an environmental induced, non-heriditary phenotype of one individual which is identical to the genotype-determined phenotype of another individual. In other words, the phenocopy induced by the environmental conditions mimics the phenotype produced by a gene. For example, a phenocopy is observed in Himalayan rabbits which have a white colored coat along with a black tail, nose, and ears when raised in moderate temperatures. However, when raised in colder climates, they develop phenotypically similar to genetically different black coated rabbits. The Himalayan rabbits exhibit black coloration of their coats, resembling the genetically encoded black rabbits. Hence in colder climates the Himalayan rabbit is a phenocopy of the black rabbit [1]. The phenocopy phenomenon can be translated and used for drug discovery processes through inhibiting a drug target with different functional modulation technologies and thereby mimicking a phenotype of interest. Inhibition can be achieved using RNA interference (RNAi), to knockdown a target, or by small molecule inhibitors (new chemical entities – NCEs) to block or inhibit the activity of the target. These modulators can be used as a particular environmental condition by treating in vitro cultured cells. Effects of the inhibition can be monitored by high-throughput RNA expression profiling and derived gene expression signatures represent either partial or exact phenocopies. Therefore, phenocopies consist of gene expression signatures caused by different pathway modulator treatments (NCE and siRNA). Subsequent analysis of the gene expression signatures will elucidate two critical issues for drug discovery: First, getting a deeper insight into a target's biology by identifying genes whose expression is transcriptionally altered after interfering with the target of interest, referred to as the TGF-β signature (on-target signature). Second, single observations for each modulator used can identify genes regulated independent of the target inhibition, referred to as the off-target signature. The TGF-β signature is independent on the used modulator and defines the biological mode of action of the target. In contrast, the off-target signature defines the mode of action for each modulator used, which has to be not necessarily limited to the inhibition of TGF-βR1 only.

So far, microarray technology has been successfully applied during the drug development process for target discovery by profiling disease models [2], for target validation by profiling alterations caused by disease-related genes [3], [4], for elucidating drug metabolism by measuring transcriptional changes of known drug metabolizing genes in rat livers or human hepatocytes [5], [6], and to address drug safety in toxicogenomics approaches [7], [8]. However, only few approaches have been tempted to fill the gap between target validation and drug metabolism and aimed to support the hit-to-lead or lead optimization processes. In fact gene expression signatures have been used to functionally annotate and characterize small molecules in yeast [9]–[11] and in mammalian cells [12]–[14]. However, these approaches mainly focused on the identification of new NCEs directed against a given target, or to build novel connections to a disease, but not to obtain an in depth analysis of the off-target effects. In our study we introduced several optimized parameters to achieve a comprehensive qualification of compounds: First, the screening platform was chosen by the use of a relevant cellular system functionally expressing the drug target and its downstream signaling. Second, various time points and concentrations were monitored. Third, siRNAs against TGF-βR1 were used as an additional target modulation technology to confirm the results obtained with the NCEs. By combining those data, the off-target signatures were used to identify the most selective NCE among the compounds tested and to detect undesirable off-target effects such as impairment of the innate immune system or of death receptor signaling. The data also allow to identify the target promiscuity of the NCE e.g. described for the multiple targeting of the anti-cancer drug Imatinib (Gleevec) or the schizophrenia drug Clozaril [15]. These polypharmacological approaches, most notably discussed in fields of cancer treatment [16], [17], cannot be faced with conventional single target-based assays but need approaches containing multi-parallel readouts for NCE characterization.

**Figure 1:**
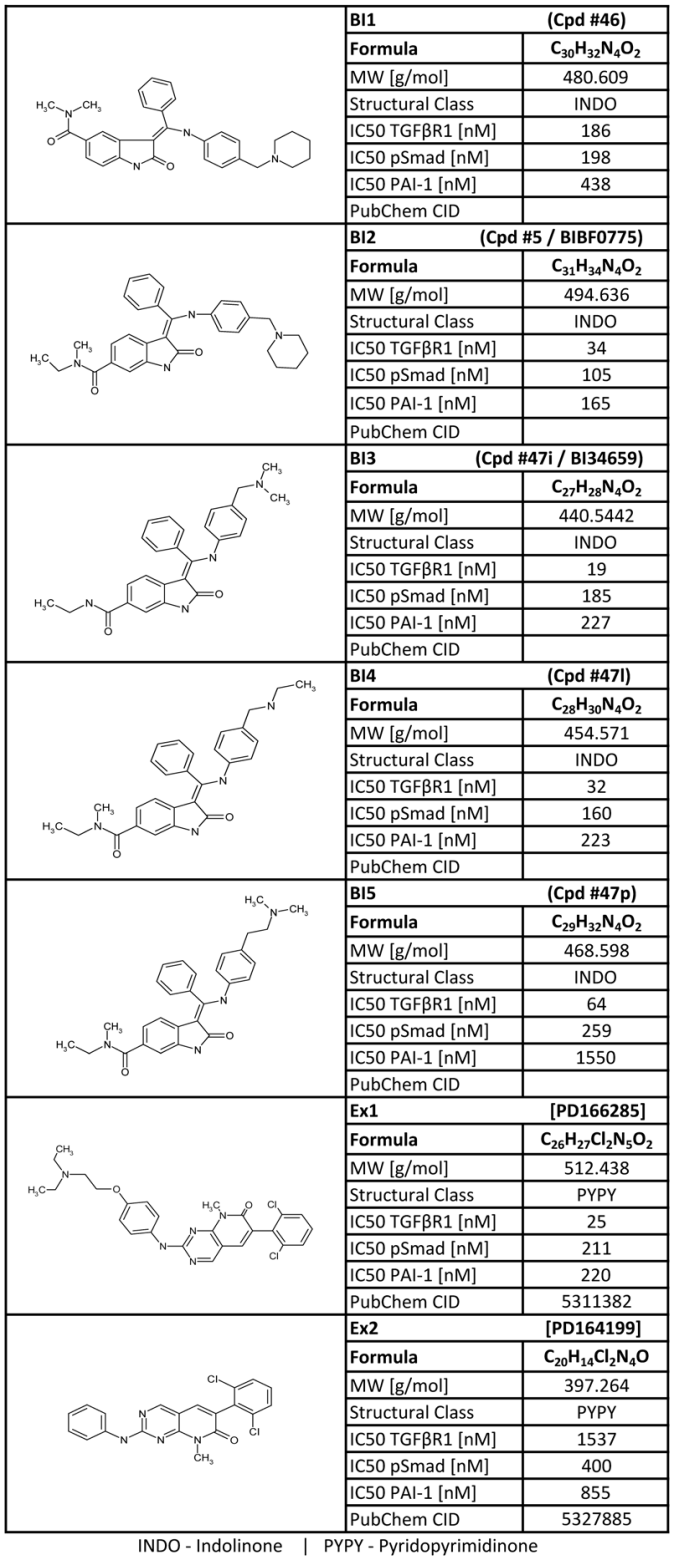
List of profiled TGF-βR1 kinase inhibitors. The chemical structures and characteristics of profiled compounds are listed. The potencies (IC50) for the inhibition of TGF-βR1 kinase, Smad2/3 phosphorylation (pSmad) and PAI-1 protein are indicated for compounds BI1 to BI5 (indolinones [INDO]) and Ex1 and Ex2 (pyridopyrimidinones [PyPy]). The PubChem CIDs are indicated. According to the chemical synthesis of the compounds (Roth et. al), the corresponding compounds identification numbers are indicated in brackets.

In this proof of concept study the phenocopy approach was applied during the lead optimization (LO) phase of our Transforming Growth Factor β Receptor I kinase (TGF-βR1) research project. 5 advanced NCEs from the project [18] (BI1-BI5, see Roth et al.) together with two competitor compounds [19] (Ex1–Ex2) were qualified (Fig. 1). TGF-β is a multifunctional cytokine with effects on cell growth, migration, adhesion, differentiation and apoptosis. Thus, malfunctions within the TGF-β signaling pathway may result in cancer, fibrosis and diverse hereditary disorders [20]–[22]. While the primary focus is in the area of cancer, there are three different therapeutic approaches under investigation dealing with antisense oligonucleotides, monoclonal antibodies (NBEs) and small molecular inhibitors (NCEs) [23].

**Figure 2:**
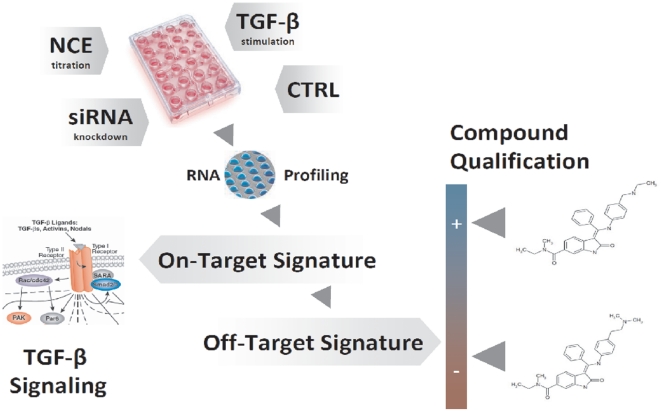
Phenocopy workflow. In vitro cultured HaCaT cells stimulated with TGF-β were treated with NCEs inhibiting the kinase activity of TGF-βR1 or with a siRNA specific against TGF-βR1. After 2 h, 4 h and 12 h total RNA was isolated for hybridization on Illumina Beadchips and expression profiles were generated. The concentration and time-dependent on-target- (TGF-β signature) as well as the off-target signatures for every NCE were obtained by bioinformatic analysis. Compounds were qualified according to their off-target signature by influencing other pathways.

In the present study, treatment of TGF-β stimulated cells with NCEs and siRNAs was monitored by high-throughput RNA expression profiling. The time-dependent TGF-β–dependent mechanism-of-action was determined and single NCE-specific and/or lead-structure-specific off-target signatures were identified (Fig. 2). The phenocopy assessment of the NCEs during the lead optimization process supplemented classical biological evaluation parameters such as potency and specificity data. It therefore allows a more integrated view on the quality of the NCEs. Ideally it can serve as a tool for ranking compounds classes or even single compounds, facilitating the decision on follow-up activities such as further vivo studies (efficacy & toxicology).

## Results

### Phenocopy Platform

To perform the phenocopy approach HaCaT cells (human keratinocytes) were cultured to analyze TGF-βR1 modulators. siRNAs and seven NCEs (Fig. 1) were used to monitor and characterize mRNA transcriptional changes upon knockdown of TGF-βR1 mRNA or inhibition of TGF-βR1 kinase activity. Subsequently, the inhibition of the TGF-β–dependent signal transduction by the selected candidates was confirmed. To cover the entire TGF-β signaling process three readouts representing early, intermediate, and late responses to TGF-β stimulation were performed. Direct downstream targets of the activated TGF-βR1 kinase are Smad2 and Smad3 proteins. Their phosphorylation initiates the intracellular signaling cascade (Fig. S1a). Therefore, as an immediate early readout a phospho-Smad2/3 ELISA was used to determine the cellular IC50 values for all seven NCEs. Additionally, to cellular IC50 values the biochemical IC50 values were obtained from kinase assays. A wide range in the inhibition of TGF-βR1, from 19 nM (BI3) to 1537nM (Ex2), was observed. A detailed list of potencies of all NCEs is provided in Fig. 1.

A well characterized downstream target of TGF-β signaling is PAI-1 (SERPINE-1) [24]. The expression of PAI-1 at mRNA levels (qRT-PCR) and at protein levels (ELISA) for TGF-β signal transduction was measured as an intermediate and a late response. An up to 70-fold up-regulation of PAI-1 mRNA was detected 6 hours after TGF-β stimulation (Fig. S1b). Subsequently, the supernatants were analyzed for PAI-1 protein expression. The expression of PAI-1 protein was delayed compared to the mRNA expression and can therefore be considered as a late response to TGF-β stimulation. The first significant increase was seen 12 hours post stimulation (Fig. S1c).

To guarantee optimal siRNA-mediated TGF-βR1 knockdown 10 commercially available siRNAs were qualified. First, knockdown efficacy was determined on mRNA level. Only 5 siRNAs (A1, D1, D2, Q3 and Q4) which led to a knockdown of more than 90% were selected for off-target profiling (Fig. S2a). Second, inhibition of downstream signaling of each selected siRNA was determined by phospho-Smad2/3 (Fig. S2b) and PAI-1 ELISA (Fig. S2c). Interestingly, although transfection of siRNA D1 resulted in the best mRNA knockdown (98%), this finding was not represented in the functional readouts. The strongest functional knockdowns were observed for siRNA A1. Finally, the off-target effects of all siRNAs were determined by microarray analysis using Illumina Beadchip technology. All deregulated genes (p-value <0.01 and |LR| ≥1) were identified for the selected five siRNAs (Fig. S3). To exclude genes from the off-target list that are relevant for the mechanism of the procedure or relevant for the TGF-βR1 biology, only those genes were selected that were uniquely deregulated by the respective siRNA. Due to its superior functional knockdown abilities (Fig. S2b) and little off-target effects siRNA A1 (Fig. S3b) was used in all further experiments.

The profiling of seven NCEs at seven different concentrations and three time points, including siRNA A1, all appropriate controls and biological triplicates for each condition resulted in an overall experimental setup of 621 samples to be submitted to array profiling.

### TGF-β signature

**Figure 3:**
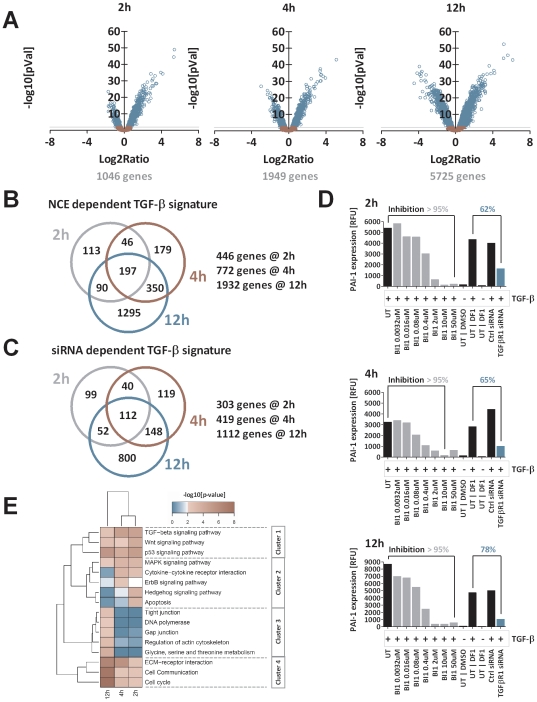
On-target Signature. The on-target signature was generated based on gene regulations upon treatment with TGF-β, TGF-βR1-kinase inhibitors (NCEs) or a siRNA. A: Volcano plots of the comparison between TGF-β stimulated and non stimulated cells at 2 h, 4 h & 12 h. Every circle represent a single transcript. The x-axis shows the log2 ratio (LR) between TGF-β stimulated vs. untreated HaCaT cells. The y-axis is scaled as negative log10 [p-value] as an indicator of significance. P-values were FDR-corrected according to Benjamini-Hochberg. Blue circled genes are significantly regulated by the stimulation with TGF-β (p-value <0.01). B: The list of non-redundant genes was filtered for a dose-dependent regulation upon NCE treatment and TGF-β stimulation.: 446, 772 and 1932 genes were identified as the NCE-dependent on target TGF-β signature after 2 h, 4 h and 12 h. C: The siRNA dependent TGF-β signature identified 307, 419 and 1112 genes which were classified as siRNA-dependent on-target TGF-β signature genes after 2 h, 4 h and 12 h. D: Expression level of PAI-1 mRNA as a surrogate marker for TGF-β signaling pathway activity after treatment with NCE B1 or siRNA. Treatment with NCE BI1 resulted in a complete knockdown of PAI-1 expression (>95%) for all time points. In contrast the siRNA A1 mediated knockdown of the TGF-β signaling only reduced PAI-1 levels partially to 62%, 65% and 78% after 2 h, 4 h and 12 h of TGF-β stimulation. E: Gene set enrichment analysis (GSEA) using KEGG gene annotation resulted in 16 significantly affected genesets/signaling pathways. Clustering of -log10[p-values] using complete linkage and manhattan distance resulted in four major clusters: immediate early affected pathways (cluster 2) permanently affected pathways with emphases at early (cluster 1) and late time points (cluster 4) or late established events (cluster 3). The color code defines the significance determined by Fisher's exact test: blue <2 – not significant; white  = 2 – significant & red >2 – highly significant).

To gain a deeper insight into the TGF-β biology we first identified genes that are regulated due to TGF-β stimulation (5 ng/ml). To unravel the time-dependent effects of TGF-β treatment HaCaT cells were stimulated with TGF-β for 2 h, 4 h and 12 h. While immediate early genes that are directly regulated by the TGF-β pathway are detected at 2 h post stimulation, more and more secondary effects linked to TGF-β signaling are found after 4 h and/or 12 h. To avoid arbitrary log ratio cut-offs we used a modulator-based approach to identify TGF-β dependent gene regulation. We applied two criteria to identify TGF-β regulated genes: First, genes that were significantly deregulated (p-value <0.01) in a basic comparison of TGF-β stimulation versus unstimulated cells were further analyzed. We found 1046, 1949 and 5725 genes (6525 non-redundant genes) to be regulated 2 h, 4 h and 12 h after stimulation (Fig. 3a). In a second step, these genes were proven to be affected dose-dependently by kinase inhibitor treatment after TGF-β stimulation. The approach allowed separating these genes from potential compound related off-target effects. All transcripts identified for each NCE were merged to a common signature of TGF-β dependent genes. This strategy allowed the identification of a common on-target signature minimized for the amount of false positive and false negative genes. The Venn diagram (Fig. 3b) depicts the number of genes that were identified after 2 h, 4 h, and 12 h of stimulation: 446 genes (2 h), 772 genes (4 h) and 1932 genes (12 h). All gene identifier annotations and regulations are listed in Table S1a.

Beyond the inhibition of the kinase activity by chemical compounds, the TGF-β pathway was also silenced by siRNA knockdown. All previously selected genes (TGF-β stim. vs. unstimulated cells, Fig. 2a) were tested to be regulated by siRNA-mediated knockdown of TGF-βR1. To exclude mechanistical effects, genes were only selected when they were regulated by siRNA A1, and not by a control siRNA (p-value <0.01). By siRNA knockdown of TGF-βR1, 303 (2 h), 419 (4 h) and 1112 (12 h) genes are identified as TGF-β dependent (Fig. 3c). Although fewer genes were identified compared to the NCE approach, the majority of genes were identified by both approaches. All gene identifier annotations and regulations are listed in Table S1b. According to the siRNA transfection procedure, a slightly different experimental setup was performed regarding cell seeding and culture conditions. That variation resulted in procedure-specific changes in gene regulation, which had to be separated from the TGF-β signature. Addressing the level of TGF-βR1 activity upon siRNA transfection, we analyzed the expression of PAI-1 as a surrogate marker for the TGF-β signaling activity. Using NCEs, we were able to inhibit the PAI-1 expression by more than 95% at all time points. In contrast, the use of siRNA A1 reduced PAI-1 levels only to 62% after 2 h of TGF-β stimulation (Fig. 3d). Despite the high efficiency of the siRNAs (A1 showed a mRNA knockdown efficiency of greater than 90%; Fig. S1a) analyzed at the time points the siRNA treatment resulted only in a partial reduction of the TGF-β signaling activity most likely due to the long half-life of the receptor protein.

Subsequently, the genes of the TGF-β signature were used to perform gene set enrichment analysis (GSEA) [25], [26]. The annotation of the Kyoto Encyclopedia of Genes and Genomes (KEGG) pathways delivered gene-sets corresponding to 201 different pathways [27], [28]. The GSEA resulted in 16 different signaling pathways which were significantly influenced upon TGF-β stimulation of HaCaT cells. The signaling pathways were clustered in four groups (Fig. 3e). Not surprisingly, the TGF-β signaling pathway itself as well as directly affected pathways like WNT and p53 signaling were significantly regulated by the treatment of TGF-β (cluster 1). In cluster 2 MAPK, cytokine, ErbB, Shh, as well as apoptosis signaling pathways are strongly affected immediately early upon TGF-β stimulation. The modulation is reduced at later time points (4 h & 12 h), when more secondary effects, such as DNA polymerase, actin cytoskeleton, amino acid metabolism, gap junction and tight junction signaling become apparent (cluster 3). The activation of these pathways in combination with the modulation of the cell cycle and cell communication activity (cluster 4) seem to be the phenotypic consequences to TGF-β stimulation of HaCaT cells. To proof the findings obtained from the KEGG analysis, we additionally used Ingenuity Pathway Analysis (Ingenuity Systems®, www.ingenuity.com) to link and group genes from the TGF-β signature. In line with the KEGG results, the analysis identified the same connections and networks containing signaling but also WNT and Erk/MAPK signaling (Fig. S4). In addition, diverse networks of genes were identified that play a role in embryonic development of different organs, but also in cellular proliferation and growth (data not shown). Having identified the TGF-β signature (on-target signature) as well as the affected pathways by GSEA, we next screened the seven NCEs for their specific off-target effects and affected pathways.

### Off-target signature

In our study, we define a phenocopy as copy of a phenotype (measured by gene expressions). Gene expressions are therefore the first step that defines the phenotype of an organism. Each compound treatment resulted in a unique gene expression signature of regulated genes. These signatures are composed of the cellular response to two different stimuli (TGF-β and NCE) and are integrated to the corresponding treatment signature. An off-target effect is defined as observed gene expression change induced by NCE treatment independent of the previously determined effects following TGF-β signal inhibition. Examples for all different types of off-targets are given in Figure S5. Thereby, elucidating the effects based on NCE treatment is more demanding since both TGF-β and off-target effects occur. Minor effects can also be observed for the interaction of the vehicle (DMSO) and the NCEs. The effects of the different stimuli overlap and also interfere with each other impeding with a clear signature dissection. The profile of a given gene may therefore be dependent on which effect prevails and thus dose-dependency might no longer be observed. In general, all regulated genes can be grouped into six classes: Pure TGF-β effects (Fig. S5a) and pure off-target effects (Fig. S5b), where genes are dose dependently regulated dependent or independent of TGF-β. Additionally, an integration of both TGF-β and off-target effects can be detected: an NCE effect can be additive (Fig. S5c) or inverse (Fig. S5d) to the effect of TGF-β. Furthermore, opposed bipolar effects for high and low dosage of the NCE mostly linked with toxicity (Fig. S5e), or common- and dose independent effects observed for all seven NCEs can be identified (Fig. S5f).

In a first approximation the NCE treatment phenotypes (phenocopies) were determined as the total of all regulated genes (p-value <0.01 and |LR| ≥1) comparing NCE treated and TGF-β stimulated cells to DMSO control treated TGF-β stimulated cells. This analysis was done separately for each of the tested compounds at each concentration. Subsequently, the different phenotypes obtained after 2 h NCE treatment were clustered to unravel similarities between the different signatures (Fig. S6). The early time point allowed focusing on primary affected genes that were altered as direct response to the treatment. Hierarchical clustering clearly revealed two major clusters separating the group of indolinones (BI1 to BI5) from the pyridopyrimidinones (Ex1 & Ex2). The fact that most obviously the specific chemotype has a major impact on differences in gene expression confirms that the classical notion of chemotypes determining biological profiles of NCEs holds true in this case. However, not only the scaffold itself but also the specific decoration of each chemotype affected gene expression. The hierarchical cluster analysis demonstrates that treatment signatures can be used to differentiate even between analogs of the same chemotype.

**Figure 4:**
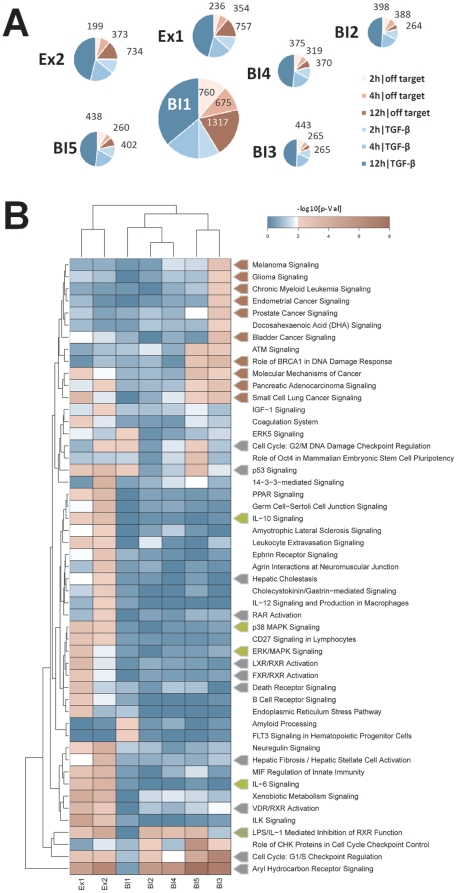
Off-target Signature. A: Every circle represents one of the seven profiled compounds. The size of each circle corresponds to the number of off-target genes (in red). On-target genes numbers are shown in blue. B: Ingenuity pathway analysis for the off-target genes of all seven NCEs after 12 hours: Clustering of the -log10[p-values] using complete linkage and manhattan distance depicts the 51 significantly ranked canonical signaling pathways. Off target genes deregulated by BI3 treatment affect almost exclusively 10 cancer signaling pathways (red arrows). Ex1 & Ex2 off-target genes play a role in 12 pathways involved in cytotoxicity or cell death (grey arrows) and in five pathways involved in inflammation (green arrows). The color code defines the significance determined by Fisher's exact test as –log10[p-value]: blue <2 – not significant; white  = 2 – significant & red >2 – highly significant.

However, the identification of a particular off-target based on this approach is difficult. Further analyses were therefore performed to extract the compounds' off-target effects from treatment signatures. As abovementioned not all off-target effects can be identified through dose dependence correlation due to overlapping, inverse and additive effects (Fig. S5). Hence off-targets can only be identified based on NCE treated samples in presence and absence of the TGF-β stimulus. All regulated genes (p-value <0.01 and |LR| ≥1) comparing compound treated cells (either 0.08 µM or 2 µM) to DMSO treated controls were selected. Genes were considered once the regulation is observed during compound treatment upon TGF-β stimulation as well as without TGF-β stimulation. Thus, we ensured to select only drug target and stimulation-independent alterations. All genes that match the criteria were allocated to the off-target signature of the NCE after 2 h, 4 h and 12 h. Based on this analysis, huge differences in the amount of off-target genes were observed. While treatment with BI1 deregulated 2752 genes at all time points, BI3, deregulated only 973 genes. Slightly more off-target genes were identified for the indolinones BI2, BI4 and BI5 (1050, 1064 and 1100). Both pyridopyrimidinones regulated 1347 (Ex1) and 1306 (Ex2) genes. The largest off-target increase over time was seen for Ex1 and Ex2 with almost four times more genes being regulated comparing the 12 h to the 2 h time point. In contrast the amount of off-targets for the five indolinones is at a maximum doubled within this period (Fig. 4a). In summary, looking at the off-target signatures in general, the indolinones appeared more favorable compared to the pyridopyrimidinones at later points in time. Among the indolinones, BI2 to BI5 deregulated fewer genes than BI1 at all points in time which was paralleled by the different kinome specificities (see Chapter ‘Kinase Profiling’). It also confirmed the structure-activity relationships described in Roth et al. [18] demonstrating that indolinones substituted in position 5 (such as BI1) showed a less favorable selectivity profile compared to indolinones substituted in position 6 (such as BI2-5). Among the indolinones, BI3 appeared to be the most attractive compound when merely looking at the off-target analysis.

### Pathway Analysis

Ingenuity Pathway Analysis was used to subsequently analyze the off-target signatures of all NCEs in order to enrich influenced signaling pathways. We found 39 (2 h), 38 (4 h) and 51 (12 h) canonical signaling pathways scored with a significant –log10[p-value] >2 (Fisher's exact test) for at least one of the NCEs (Fig. 4b). Pathway analysis again separated the indolinones from the pyridopyrimidinones indicating that both series share not only a common mode of action like TGF-β inhibition, but also generate a distinct affection of other pathways by their specific off-target function. In accordance with the structure-activity findings mentioned before, BI1 stands apart from the four other indolinones with 5 significantly ranked pathways and the smallest overlap with the other indolinones. BI3 affects 15 signaling pathways and almost exclusively regulates genes involved in different cancer pathways. The indolinones BI2 and BI4 regulated genes that are significantly enriched in only four (BI2) and two (BI4) signaling pathways, respectively. However, pathways such as the Aryl Hydrocarbon Receptor Signaling and the LPS/IL-1 mediated inhibition of RXR function are also significantly ranked high for up to six compounds indicating a more general effect like a xenobiotic response to NCE treatment rather than a true compound specific effect. The highest numbers of significantly influenced pathways are found for the two pyridopyrimidinones with 29 (Ex1) and 24 (Ex2). Additionally, genes involved in 30 out of the 51 signaling pathways are exclusively regulated by Ex1 or Ex2 treatment.

Interestingly, 13 out of the 51 identified pathways are known mediators of toxicity and cell death. These 13 pathways reach highest significance scores for either Ex1 or Ex2 with eight being solely affected by the two pyridopyrimidinones indicating a cytotoxic mode of action for both of them. Besides cytotoxicity these two NCEs deregulate genes involved in inflammatory processes like IL-6 signaling, ERK/MAPK signaling, and p38 MAPK signaling (Fig. 4b).

Results from the pathway analysis strongly implied different induced phenotypes after treatment with specific NCEs. However, pathway analysis tools only generate hypotheses and their proof of biological relevance must be verified. To address the accuracy of the pathway analysis we aimed to confirm the in silico generated hypotheses by experimental laboratory data.

### Cytotoxicity and Cell Death

**Figure 5:**
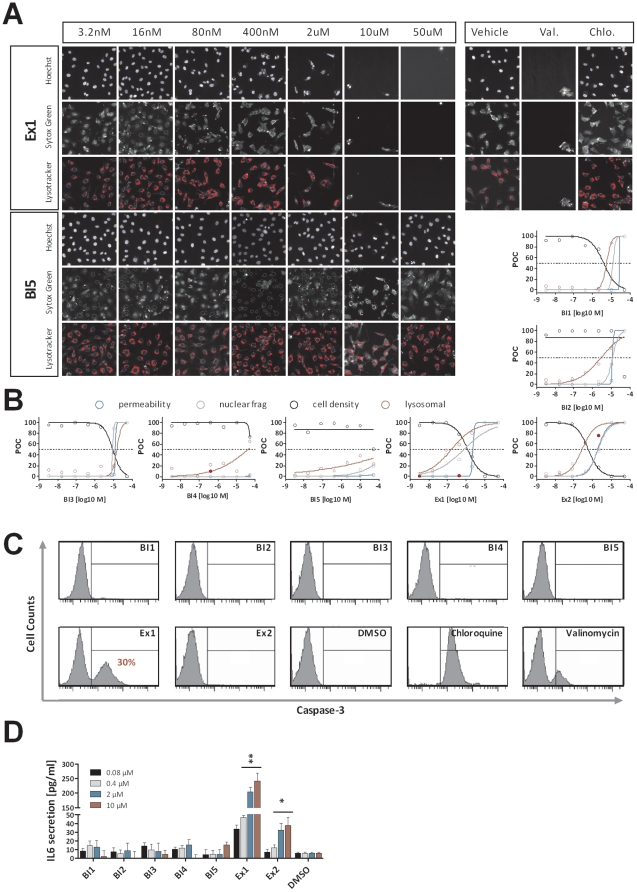
Wet laboratory validation of in silico results. Functional assays were used to validate the predictions derived from the pathway analysis. Cellomics high content screens analyzing cytotoxic parameters and Caspase 3 activation assays were performed to test for cytotoxicity and cell death. IL-6 secretion was analyzed as surrogate marker for pro-inflammatory processes. A: High content screen images of HaCaT cells treated with increasing concentrations (3.2 nM – 50 µM) of Ex1 and BI5 including DMSO (negative control) or 100 µM Valinomycin and 10 µM Chloroquine (positive control) for 24 h. Subsequent staining with Hoechst dye, Sytox Green and Lysotracker Red determined cell density, nuclear fragmentation, permeability and lysosomal mass per cell illustrated a strong decrease of cell density and an increase of nuclear fragmentation and lysosomal mass for Ex1 but not for BI5 treated cells. B: Results of concentration response experiments for all seven NCEs for cell density (black) nuclear fragmentation (grey), permeability (blue) and lysosomal mass (red) obtained from Cellomics high content screen analysis. Values of NCE treated cells are compared to DMSO treated cells and shown as percent of control (POC). Outlier data point are shown as filled red circles C: HaCaT cells were treated with 2 µM of each NCE and incubated for 24 h. Caspase-3 activation was analyzed. A significant signal was identified only after treatment with Ex1 (30%) or with Valinomycin (15%) and Chloroquine (100%). D: HaCaT cells were treated with increasing NCE concentrations and DMSO for 12 h. IL-6 levels were app. 25-fold increased after Ex1 treatment and 5-fold increased after Ex2 treatment compared to DMSO treated cells. Student t-test was used to calculate the significance compared to DMSO treated cells (*<0.01 & **<0.001). All error bars indicate the standard deviation of n = 3.

According to the expression data, both pyridopyrimidinones (Ex1 & Ex2) are involved in processes such as cell death and inflammation. To investigate several cytotoxicity parameters, we performed high content screen analysis using the high-capacity automated fluorescence imaging platform from Cellomics. HaCaT cells were incubated with increasing compound concentrations (3.2 nM – 50 µM) for 24 h. Subsequently the cells were stained with cytotoxicity cocktails and images were acquired and analyzed on the Cellomics ArrayScan II. Cells were stained using i) Hoechst DNA dye to count cell density and investigate nuclear condensation and fragmentation, ii) LysoTracker Red to analyze the amount of lysosomes per cell as an early marker for cytotoxicity, iii) Sytox Green as a membrane impermeable dye to detect loss of membrane integrity as late event for cytotoxicity (Fig. 5a). According to the aforementioned pathway analysis the highest toxicity is observed by treatment with Ex1 and Ex2. Cell density is strongly decreased to less than 10% of control. Nuclear fragmentation, lysosomal mass per cell and membrane permeability are increased by 100% for both pyridopyrimidinones and even lower concentrations of Ex2 were sufficient to raise the membrane permeability of more than 50% of the control. Treatment with the indolinones resulted in mild toxicity effects for the treatment with BI1, BI2 and BI3 at high concentrations and almost no toxicity for BI4 and BI5 (Fig. 5b).

To analyze the mode of cell death, we performed Caspase-3 activation assays to distinguish between apoptosis and necrosis, since activation of this executioner caspase is a clear marker for apoptotic cell death. Again, HaCaT cells were treated with 2 µM of each compound for 24 hours and subsequently Caspase-3 activation was detected using a Caspase-3 Detection Kit (Calbiochem) and quantified by flow cytometry. Among all NCEs tested, only treatment with Ex1 resulted in an activation of Caspase-3 with approximately 30% positive cells (Fig. 5c). This is in line with the results of the pathway analysis in which only the off-target signature of Ex1 exceeded the significance threshold for Death Receptor Signaling after 4 h (data not shown) and was further increased after 12 h (Fig. 5b). This clearly demonstrates that it was not only possible to predict the compound's cytotoxicity based on mRNA profiles but also its apoptotic mode of action.

Pathway analysis labeled the pyridopyrimidinones for cell death induction, but also for affection of inflammatory mechanisms. To proof this prediction, HaCaT cells treated with each compound were analyzed for the induction of pro-inflammatory cytokines (IL1-β, TNF-α, IL-8 and IL-6). While no significant alteration in release of IL1-β, TNF-α, and IL-8 was observed, we could demonstrate that IL-6 levels were dose-dependently increased after treatment with the pyridopyrimidinones Ex1 and Ex2. Compared to DMSO treated control cells 10 µM of Ex2 increased IL-6 secretion by factor 5 and Ex1 treatment at the same concentration even resulted in a 25-fold increase (Fig. 5d). In summary, the indolinones looked more favorable in this evaluation compared to the pyridopyrimidinones.

### Kinase Profiling

**Figure 6:**
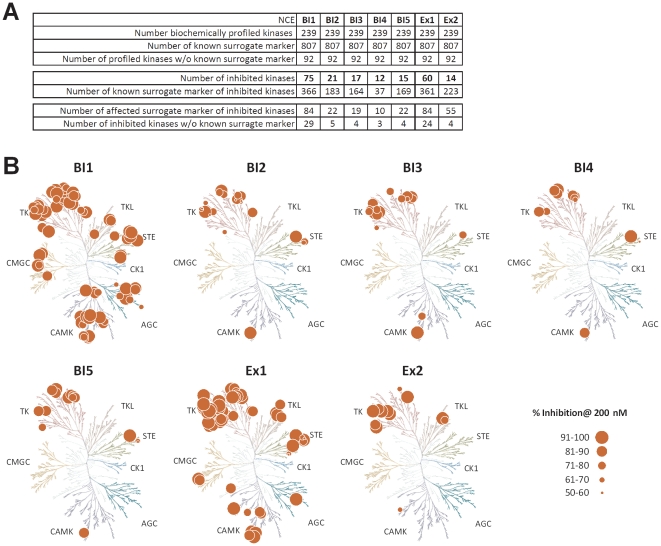
Kinase Selectivity. A: Each compound was profiled against a panel of 239 protein kinases and the number of kinases inhibited by each compound is shown (Table S3). No surrogate marker (e.g. literature known downstream target of a given kinase) were identified for 92 enzymes, whereas 807 surrogate markers were identified for 147 of the enzymes. Based on the kinases inhibition profile of each compound, the expression of these kinase in HaCaT cells and the availability of surrogate markers the number of potentially effected surrogate marker genes was predicted. B: Kinome dendrograms used for visualization were shown with permission from Cell Signaling Technology, Inc. (http://www.cellsignal.com). Human kinome dendrograms showing the NCEs' kinase specificity profiles. Circle size corresponds to the percentages of inhibition of the kinase at 200 nM concentration. AGC – Containing PKA, PKG, PKC families; CAMK – Calcium/calmodulin-dependent protein kinase; CK1 – Casein kinase 1; CMGC – Containing CDK, MAPK, GSK3, CLK families; STE – Homologs of yeast Sterile 7, Sterile 11, Sterile 20 kinases; TK – Tyrosine kinase; TKL – Tyrosine kinase-like.

One pivotal issue of kinase inhibitors is cross-reactivity with other kinases which may contribute one source of off-targets. In vitro kinase profiling is the state of the art method to examine selectivity of kinase inhibitors. We used the Ingenuity Pathway Analysis database to extract the literature described downstream targets for 239 in HaCaT cells expressed kinases. For 147 kinases 807 non-redundant downstream targets had been described and annotated. The downstream targets were used as surrogate markers and overlaid with the NCE's off-target lists to assign off-target genes to off-target kinases (Fig. S7). The correlation of the in vitro predicted kinase inhibition with the off-targets requires some criteria to be fulfilled: i) the kinase has to be expressed in the cellular system, ii) the signaling pathway must be functional, which iii) depends on the availability of appropriate ligands in the in vitro system. As the cells in our study had been starved for compound and/or TGF-β profiling, these criteria might have been only partially met. Finally, iv) surrogate markers had to be described. The regulation of surrogate markers for kinase inhibition was used to predict the activity of upstream acting kinases for each of the tested compounds. To proof the predictivity of our model, we tested all compounds against 239 kinases available in the biochemical SelectScreenTM Kinase Profiling (Invitrogen) at concentrations of 2 µM and 200 nM. All kinases inhibited by at least 90% at 2 µM conc. and additionally by at least 50% at 200 nM were selected as off-target kinases (Table S3). Most off-target kinases were identified for BI1 (75) and Ex1 (60). All other NCEs showed a higher selectivity with only 21 (BI2), 17 (BI3), 12 (BI4), 15 (BI5) and 14 (Ex2) kinases inhibited by the respective compound (Fig. 6b). For BI1, 84 (22.9%) of the 366 known surrogate marker genes were found to be regulated. A comparably good ratio was also identified for the two pyridopyrimidinones with 84 (23.2%) out of 361 (Ex1) and 55 (24.7%) out of 223 (Ex2). A summary of data is shown in Figure 6a and 6b. Integrating all criteria, the identification of kinase selectivities was limited, as shown for BI2 in figure S7. Although various annotated surrogate marker genes were identified as off-targets, a clear association to a specifically upstream-acting kinase was often not possible since too many surrogate markers have been redundantly identified to act downstream of several receptor kinases (Fig. S7). The cellular system might be optimized in regard to the addition of receptor ligands, but it will not replace testing NCEs in biochemical assays for kinase selectivity.

## Discussion

Since the approval of Imatinib (Gleevec) in 2001, the first marketed kinase inhibitor, many additional kinase inhibitors have been advanced into clinical development. The most advanced kinase programs in research and development are aimed at the treatment of various cancers. However, additional therapeutic applications like immunological, metabolic-, or infectious diseases and also the treatment of central nervous system disorders by kinase inhibitors are under investigation [29]–[31]. During the optimization of kinase inhibitors one often has to cope with challenges like the improvement of kinase selectivity [30], [32]. In combination with the overall high attrition rates of new drug candidates [33] there is a need for new strategies that support and optimize the drug discovery process.

**Figure 7:**
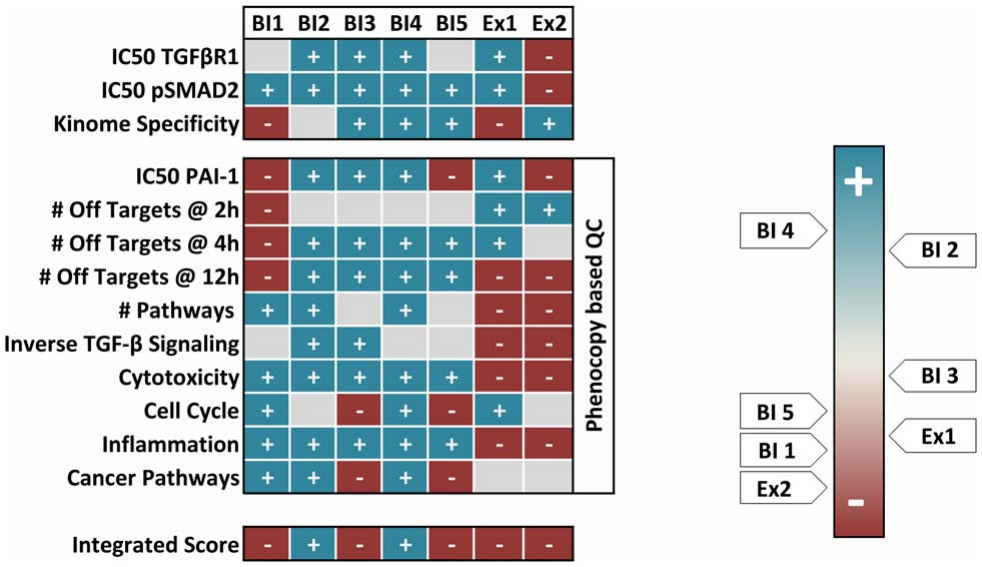
NCE ranking. Quality parameters used to gauge the seven NCEs. The phenocopy strategy introduces ten additional parameters dealing with potency, off-target numbers and affected pathways. NCEs are ranked from blue (good) to red (bad). Integration of all parameter scores identifies BI4 and BI2 as superior to all other NCEs.

So far, the in vitro biological evaluation of NCEs was often based on biochemical and cellular potencies, as well as on the selectivity of the respective NCE. This limited view may result in wrong decisions for further time and cost consuming processes, such as in vivo experiments. In the present study, we have established a workflow to alleviate the lead identification and optimization of NCEs in general and kinase inhibitors in particular by elucidating the mechanism of action of both the target and the NCE. Thereby, more knowledge about drug candidates is obtained at an early stage of drug discovery and several new categories for their qualification are available (Fig. 7).

By evaluating of TGF-βR1 inhibitors, we were able to clearly differentiate the indolinone chemotype from the pyridopyrimidinones in several parameters. Furthermore, even within the indolinone cluster differences between compounds with different decorations were identified. Besides the detection of off-targets and biomarkers, this strategy can also help to interpret the identified off-target effects and offers the possibility to assign the regulated genes to relevant biological processes and networks. This can be handled in a flexible format by the respective scientist by defining context relevant processes or just by prioritizing the compounds in terms of the absolute number of affected processes or pathways. In terms of TGF-β inhibition for instance one goal is to reduce inflammation processes triggered by this cytokine [34]. Thus, the predicted and confirmed pro-inflammatory properties of both pyridopyrimidinones (Fig. 4b & Fig. 5d) are the opposite of the desired effect making both NCEs inferior to the indolinones for the treatment of fibrotic diseases and/or cancer. Furthermore relevant and unwanted processes are regulation of growth and proliferation and obviously induction of cell death (Fig. 5b). Finally, the combination of TGF-β and off-target signatures revealed that some compounds regulate genes inverse to the desired therapeutic effect (Fig. S5d). This can potentially affect the efficacy of the treatment with this NCE. Particularly both pyridopyrimidinones regulated 217 (Ex1) and 317 (Ex2) TGF-β dependent genes in the opposite direction to the desirable treatment effect. In contrast the indolinones only affected a lower number (BI3: 42; BI2: 57, BI1: 70; BI5: 81; BI4: 101) of these genes.

According to the ten introduced phenocopy criteria, a couple of compounds revealed liabilities through down-stream inhibition of PAI-1 transcription (BI1, BI5 & Ex2), at the regulation of off-target genes per se (BI1, Ex1 & Ex2), at the affection of inverse TGF-β signaling (Ex1 & Ex2), at the induction of cell death (Ex1 & Ex2), at acting as pro-inflammatory stimuli (Ex1 & Ex2) and as promoting cellular growth and induction of cancer pathways (BI3 & BI5) (Fig. 7). An integration of all obtained information recommends the use of BI4 and BI2 for further optimization due to superior overall performance of these two drug candidates.

Furthermore, our data strongly indicates that off-target effects do not only derive from additionally inhibited kinases in line with the fact that within the human genome over 2000 other nucleotide-dependent enzymes can be found [35] which potentially may be affected by NCEs blocking an ATP binding site. In addition, identification of bioactive compounds revealed a high degree of promiscuity for kinases inhibitors with GPCRs and phosphodiesterases [36]. Hence an approach using the phenocopy strategy will deliver a wider view on the NCEs' selectivity. Such a strategy will also help to accumulate an iterative knowledge about both the drug candidates itself and the structural classes. The drug candidates' off-target signature can be overlaid with other databases containing drug-dependent gene signatures like the Connectivity Map [13]. Integration of additional data sources will further characterize the NCEs by flagging them for potential side effects and the identification of desirable pharmacology profiles or even find a repositioning idea for another indication.

Not only off-target signatures but also on-target signatures can help to support the drug discovery process. On the one hand, these signatures can be overlaid with known disease signatures in order to annotate the targets contribution to the state of disease. On the other hand, it can be used to identify potent biomarkers for efficacy of the treatment and to support the clinical biomarker assay development process. This is especially important if the target's biology is not as well characterized as for TGF-βR1. However, using the phenocopy strategy we were able to significantly increase the amount of known TGF-β regulated genes by several hundred compared to earlier studies [37]–[41].

Neither the complexity of a living organism nor a disease state can be entirely represented by profiling of a single cell line. Nevertheless, the phenocopy strategy demonstrates one possibility to significantly alleviate the drug discovery process at an early stage. Comparing such an approach to classical toxicology testing or toxicogenomics studies, the phenocopy strategy offers a couple of advantages: it addresses on- and off-target effects and is able to differentiate between target-related vs. compound-related events. This differentiation is only possible when a couple of compounds of different compound classes will be investigated. Due to costs and capacities, the analysis of a certain number of compounds can only be run in vitro. Although cellular systems cannot replace in vivo studies, they show less variability, guarantee the expression and signaling of the target protein and are less cost and time consuming.

The phenocopy approach offers an opportunity to qualify and rank compound classes and single compounds early during hit-to-lead and lead optimization processes, which will subsequently reduce the attrition rates later on, e.g. during toxicological assessment of the development candidates. However, the addition of new technologies and checkpoints like phenocopy is contributing to the ever-rising costs of getting innovative medicine to the market. But nevertheless, the assembly of workflows of successfully used tools during early lead generation processes will become crucial for the discovery of novel quality of entities in a changing pharmaceutical industry. One useful tool is the phenocopy principle, where external stimuli like the climate of the environment for the Himalayan Rabbit or like NCEs, siRNAs, antibodies or aptameres for the inhibition of a cellular process is committing a certain phenotype. By investing in qualification of NCEs during the early drug discovery process, later on the attrition rate during development phases will be reduced. Indirectly, this investment will reduce the overall cost for developing innovative medicine.

## Methods

### Cell culture, NCE treatment and siRNA transfection

HaCaT cells were cultured under standard conditions [42]. Cells were seeded in 96-well (ELISA) or in 24-well (RNA expression profiling) plates and grown overnight to a confluence of approximately 70%. Cells were starved for 3 h in DMEM containing no FCS. Cells were pre-incubated with increasing NCE concentrations (0.0032, 0.016, 0.08, 0.4, 2, 10, 50 µM) for 15 min and subsequently stimulated with 5 ng/ml of TGF-β1 (R&D Systems) and incubated for the indicated time points.

10 TGFβR1-specific siRNAs were purchased from Ambion, Dharmacon or Qiagen and a nonsense control siRNA was purchased from Dharmacon. All siRNAs were prepared according to manufacturer's instructions. For transfection experiments cells were seeded in 24-well plates and grown overnight to a confluency of 50–70%. siRNAs were transfected at a final medium concentration of 20 nM. Cells were transfected using Dharmacon's DharmaFECT1 reagent. 24 h post transfection, the medium was replaced. 48 h after transfection cells were washed with PBS and lysed using RLT buffer (Qiagen).

### RNA extraction

RNA isolation was carried out using a MagMAX™ Express-96 Magnetic Particle Processor (Ambio) and the MagMAX™-96 Total RNA Isolation Kit (Ambio) according to the manufacturer's protocol. Total RNA concentration was quantified by fluorescence measurement using SYBR Green II (Invitrogen) and a Synergy HT reader (BioTek) as previously described [43]. The RNA quality was characterized by the quotient of the 28S to 18S ribosomal RNA electropherogram peak using an Agilent 2100 bioanalyzer and the RNA Nano Chip (Agilent).

### Amplification, labeling and Beadchip hybridization of RNA samples

Illumina TotalPrep RNA Amplification Kit (Ambion) was used to transcribe 200 ng toRNA according to the manufacture's recommendation. A total of 700 ng of cRNA was hybridized at 58°C for 16 h to the Illumina HumanHT-12 Expression Beadchips (Illumina). Beadchips were scanned using an Illumina BeadArray Reader and the Bead Scan Software (Illumina).

### Data processing

Data has been processed with BeadStudio version 3.0 and the R Language and Environment for Statistical Computing (R) 2.7.0 [44], [45] in combination with Bioconductor 2.2. [46]. The Bioconductor lumi package [47] has been used for quality control and normalization. The data has been log2 transformed and normalized using robust spline normalization (rsn). Linear models (Bioconductor package limma) [48] were used to calculate log2 ratios, the resulting p-values were FDR-corrected [49]. The raw data of 640 Illumina beadchips are accessible as MAIME-compliant entry at Array Express (E-MTAB-265). A fully detailed description of the normalization methods was recently published by Schmid et al [50].

### TGF-β signature

To define genes deregulated by TGF-β signaling, three sequential filtering steps were applied for each time point separately: 1) significant difference between TGF-β stimulated and unstimulated cells, 2) significant deregulation by at least one compound concentration, 3) dose dependent deregulation (R package IsoGene [51]). For each time point the probes that passed all three filters are pooled to the final TGF-β signature.

### Off-target signature

To detect transcripts that are deregulated due to off-target effects of the compounds unstimulated cells (wotgf class) as well as TGF-β stimulated cells (tgf class) were considered for compound concentrations 0.08 and 2 µM and the respective controls. Transcripts that are up/down regulated by either compound treatment (wotgfup/wotgfdown) or by TGF-β stimulation together with compound treatment (tgfup/ tgfdown) were detected based on all pair wise comparisons. **The off-target signature is composed of transcripts for which (tgf_up_ ∧ wotgf_up_) ∨ (tgf_down_ ∧ wotgf_down_) ∨ (tgf_up_ ∧ wotgf_down_) ∨ (tgf_down_ ∧ wotgf_up_). For a more detailed explanation, see Methods S1.** All off-target genes are listed as supplemental data in Table S2.

### Ingenuity Pathway Analysis (IPA) and Gene Set Enrichment Analysis (GSEA)

Based on the on- and off-target signatures, standard IPAs were used to generate networks and perform GSEA using Fisher's exact test for canonical pathways defined by the Ingenuity Knowledge Base.

Additionally, GSEA for the on-target signatures was conducted using Fisher's exact test based on gene sets defined by KEGG pathways as annotated by the Bioconductor package KEGG.db version 2.2.0.

The p-values calculated based on Fisher's exact test were clustered using manhattan distance and complete linkage.

### High content screen Cellomics

The high-content cytotoxicity assay 1 was performed according to the manufacturer's instructions (ThermoFisher Cellomics). Briefly, HaCaT cells were cultured overnight in black 96-well plates, incubated for 24h with each NCE at the indicated concentrations and stained with cytotoxicity cocktail. Cells were fixed, washed and scanned on the Cellomics ArrayScan II platform. Images were analyzed with the Cell Health image analysis algorithm. Cytotoxicity indices were calculated for each of the four parameters to indicate the percentage of cells outside of the normal range which was defined using a vehicle-treated reference cell population.

### Caspase-3 Assay

Cells were seeded in 6-well plates and grown overnight to a confluence of approximately 70% before they were treated with 2 µM of each NCE and incubated for 24 hours. Caspase-3 activity was quantified using Facs Canto (BD Biosciences) and the Caspase-3 Detection Kit (Calbiochem) according to the manufacturer's instruction.

### ELISA analysis of IL1-β, TNF-α, IL-8 and IL-6

To analyze the expression of these four cytokines cells were treated with NCEs at the indicated concentrations and incubated at 37°C for 12 h. Supernatants were analyzed using a Mesoscale Discovery muliplex ELISA System (MSD) for detection according to the manufacturer's instruction.

### In vitro kinase profiling

The SelectScreenTM kinase Profiling Service was performed (Invitrogen) to indentify the compound selectivity against 239 kinases. Single-point kinase inhibitory activities of each compound at 2 µM and 0.2 µM were measured at 100 µM or Km ATP concentration. Downstream targets of the identified off-target kinases were manually extracted from Ingenuity's Knowledge Base and overlaid with the NCE off-targets for comparisons.

## Supporting Information

Figure S1 Phenocopy platform. Three readouts representing early (Smad2/3 phosphorylation), intermediate (PAI-1 mRNA) and late (PAI-1 protein) responses to TGF-β stimulation were performed. a: phospho-Smad2/3 ELISA. This assay showed a significant increase of Smad2/3 phosphorylation 15 minutes after stimulation with TGF-β. Phosphorylation is further enhanced after 30 and 60 minutes and remains stable for further 60 minutes. b: PAI-1 mRNA. Elevated PAI-1 expression was demonstrated by qRT-PCR after TGF-β stimulation in a dose- and time-dependent manner. c: PAI-protein. The supernatants were analyzed with a PAI-1 ELISA for protein expression. The first significant increase was observed 12 hours post stimulation. Subsequently, PAI-1 further accumulated in a concentration-dependent manner. All results are representative of three independent experiments. Student t-test was used to calculate the significance compared to unstimulated cells (*< 0.01 & **<0.001). All error bars indicate the standard deviation of n = 3.
(0.08 MB PDF)

Figure S2 siRNA validation and qualification. A: siRNA knock-down efficiency was measured by Taqman RT-PCR 48h post transfection. 10 different commercially available siRNAs (A - Ambion, D - Dharmacon & Q - Qiagen) were used. B and C: siRNAs with the best knockdown efficacy (A1, D1, D2, Q3 & Q4), as well as the untreated control (UT) were analyzed for functional blockade of TGF-β signaling determined by inhibition of p-Smad2/3 (b, p-Smad2/3 ELISA) or PAI-1 protein (c: PAI-1 ELISA). All error bars indicate the standard deviation of n = 3.
(0.08 MB PDF)

Figure S3 siRNA off-target effects. Volcano plots for siRNAs A1, D1, D2, Q3 & Q4. Total RNAs of biological triplicates were isolated post siRNA transfection and were hybridized to Illumina Beadchips. The off-target effects were analyzed by volcano plots. Each circle represents a single gene of the human genome. The x-axis depicts the log2 ratio (LR) between each siRNA and untreated cells. The y-axis is scaled as -log10[p-value] (Student t-test) as a indicator of significance. An off-target is defined to have a |LR|≥1 and a -log10 [p-value] > 2. a: CTRL siRNA vs. untreated CTRL revealed no off-target effects. The siRNAs A1 revealed 22 genes to be deregulated (b), the siRNA D1 - 8 genes (c), the siRNA D2 - 25 genes (d), the siRNA Q3 - 58 genes (e) & the siRNA Q4 - 42 genes (f).
(1.20 MB PDF)

Figure S4 Ingenuity on-target Analysis. Networks of interacting and regulated molecules from the on-target signature as generated by Ingenuity Pathway Analysis. Molecules are represented as nodes and the biological relationship between two nodes is represented as an edge (line). All edges are supported by at least 1 literature reference. The intensity of the node color indicates the degree of up- (red) or down- (green) regulation. Nodes are displayed using various shapes that represent the functional class of the gene product. a: A network of molecules directly related to the canonical TGF- β signaling pathway containing genes involved in cell signaling, connective tissue development and function and in skeletal tissue development and function. b: A network of molecules of the WNT and the Erk/MAPK signaling pathways containing genes responsible for organ-, tissue and cellulare development.
(0.48 MB PDF)

Figure S5 Case profile definition. NCE treatment and TGF-β stimulation resulted in six different cases profiles of gene regulation: representative examples for on-target effects triggered by TGF-β (a); Off-target effects triggered by a NCE (b); Integrated effects for on and off-targets in an additive (c), inverse (d) and bipolar (e) manner; common off-target effects induced by all 7 NCEs in a dose-independent manner (f).
(0.12 MB PDF)

Figure S6 Hierarchical Clustering. Hierarchical clustering of 4314 significant deregulated genes (|LR| ≥ 1 & p-value < 0.01) after NCE treatment and TGF-β stimulation for 2h in HaCaT cells. The expression patterns of the different NCE treated cells reveal several intersections in gene regulation. The five indolinones (BI1-BI5) are grouped and separated from the pyridopyrimidinones (Ex1 & Ex2). Expression patterns are grouped in high vs. low dose fractions. The indolinone BI1 separates from the other class members, which can be further divided into two subgroups containing BI2 and BI3 and BI4 and BI5, respectively. Blue indicates decreased expression relative to untreated cells, red indicates increased expression.
(1.50 MB PDF)

Figure S7 In silico prediction of kinase hits. Projections of BI2-specifically regulated kinase surrogate marker genes. Indicated are all 21 inhibited kinases: 3 kinases with no affection of known surrogate marker genes (c'), 4 kinases of which no surrogate markers are described (c') and 14 kinases with de-regulated surrogate markers genes (blue line  =  transcriptional down-regulation, red line  =  transcriptional up-regulation, red box  =  in silico predicted and biochemically confirmed BI2-specific kinase hits).
(0.43 MB PDF)

Table S1 TGF-β Signature. Listed are genes significantly regulated upon TGF-β stimulation: (a) NCE treatment, (b) siRNA treatment.
(1.02 MB XLS)

Table S2 Off-target Signatures. NCE dependent Off-target Signatures identified upon 2h, 4h and 12h stimulation
(2.33 MB XLS)

Table S3 Each compound was profiled against a panel of 239 kinases. The kinases inhibited by each compound at 2μM and 200 nM are listed.
(0.07 MB PDF)

Methods S1 (0.08 MB DOC)
