# Supplementary figures and images for: Phenocopy – A Strategy to Qualify Chemical Compounds during Hit-to-Lead and/or Lead Optimization
Source: PLoS One. 2010 Dec 10;5(12):e14272. doi: 10.1371/journal.pone.0014272 (PMC3000806; doi:10.1371/journal.pone.0014272)

Figure S1

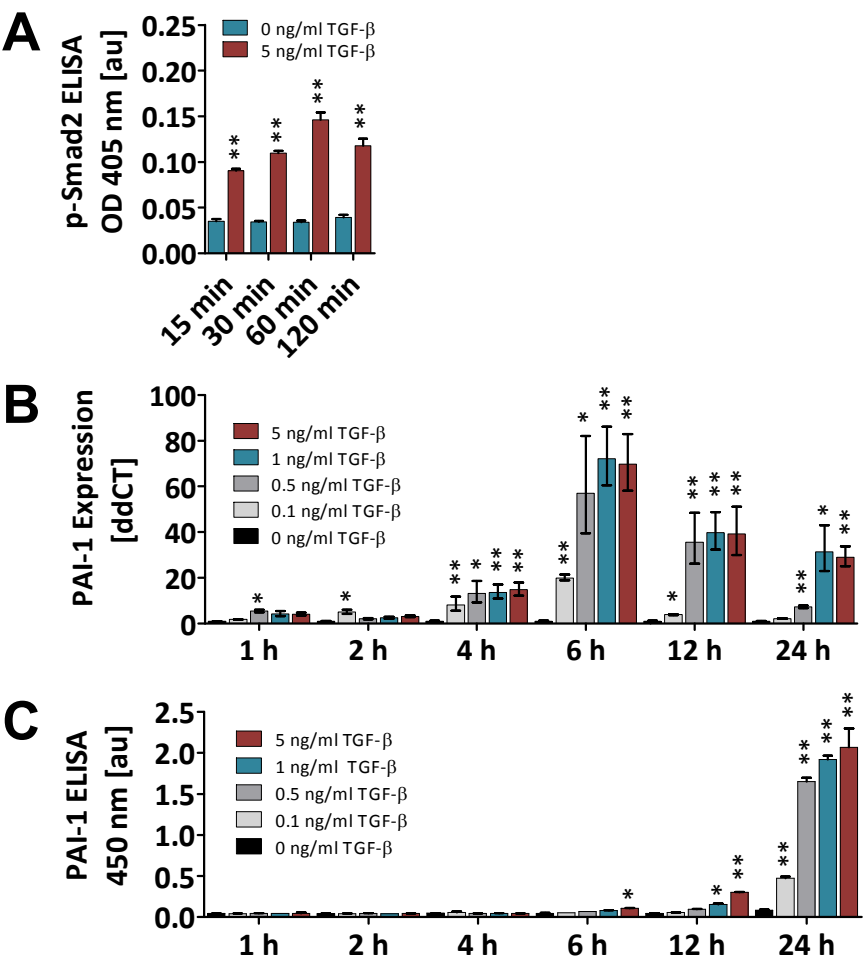

Supplement: Figure S1 — Phenocopy platform. Three readouts representing early (Smad2/3 phosphorylation), intermediate (PAI-1 mRNA) and late (PAI-1 protein) responses to TGF-β stimulation were performed. a: phospho-Smad2/3 ELISA. This assay showed a significant increase of Smad2/3 phosphorylation 15 minutes after stimulation with TGF-β. Phosphorylation is further enhanced after 30 and 60 minutes and remains stable for further 60 minutes. b: PAI-1 mRNA. Elevated PAI-1 expression was demonstrated by qRT-PCR after TGF-β stimulation in a dose- and time-dependent manner. c: PAI-protein. The supernatants were analyzed with a PAI-1 ELISA for protein expression. The first significant increase was observed 12 hours post stimulation. Subsequently, PAI-1 further accumulated in a concentration-dependent manner. All results are representative of three independent experiments. Student t-test was used to calculate the significance compared to unstimulated cells (*< 0.01 & **<0.001). All error bars indicate the standard deviation of n = 3. (0.08 MB PDF) [file pone.0014272.s001.pdf]

Figure S2

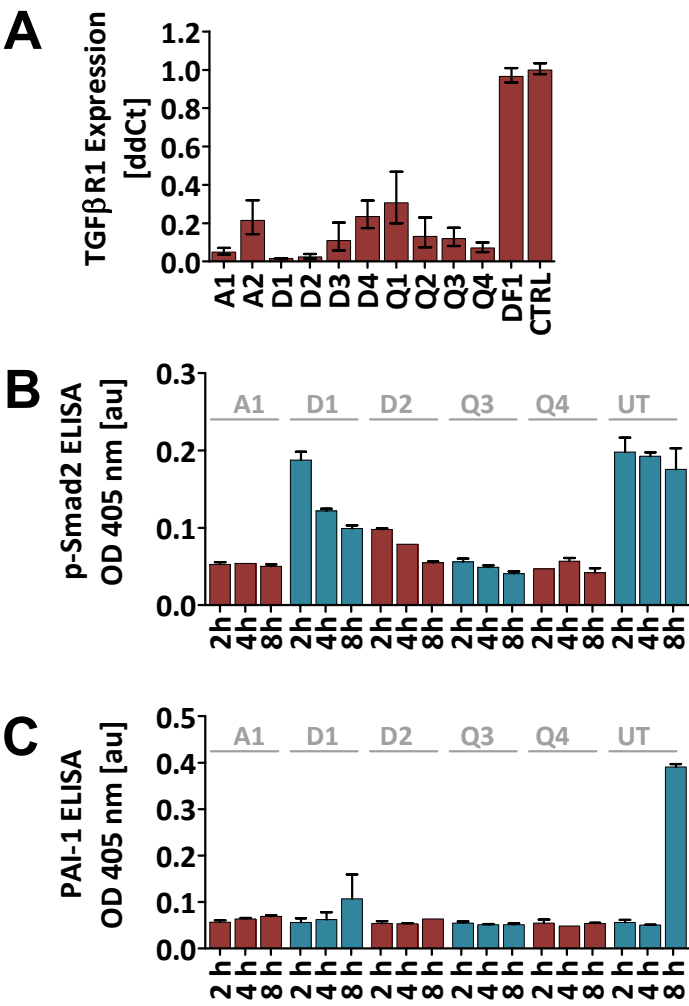

Supplement: Figure S2 — siRNA validation and qualification. A: siRNA knock-down efficiency was measured by Taqman RT-PCR 48h post transfection. 10 different commercially available siRNAs (A - Ambion, D - Dharmacon & Q - Qiagen) were used. B and C: siRNAs with the best knockdown efficacy (A1, D1, D2, Q3 & Q4), as well as the untreated control (UT) were analyzed for functional blockade of TGF-β signaling determined by inhibition of p-Smad2/3 (b, p-Smad2/3 ELISA) or PAI-1 protein (c: PAI-1 ELISA). All error bars indicate the standard deviation of n = 3. (0.08 MB PDF) [file pone.0014272.s002.pdf]

Figure S3

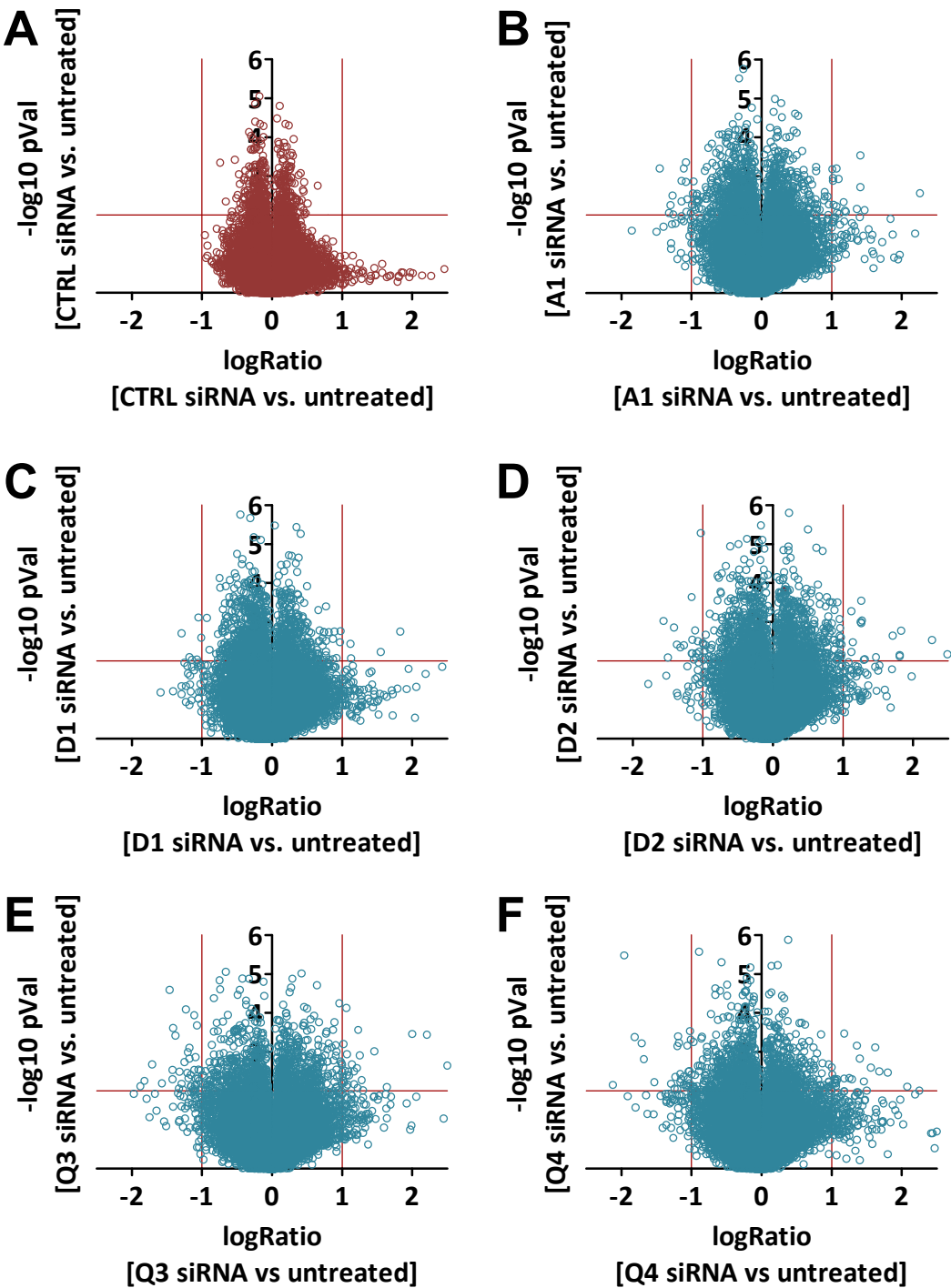

Supplement: Figure S3 — siRNA off-target effects. Volcano plots for siRNAs A1, D1, D2, Q3 & Q4. Total RNAs of biological triplicates were isolated post siRNA transfection and were hybridized to Illumina Beadchips. The off-target effects were analyzed by volcano plots. Each circle represents a single gene of the human genome. The x-axis depicts the log2 ratio (LR) between each siRNA and untreated cells. The y-axis is scaled as -log10[p-value] (Student t-test) as a indicator of significance. An off-target is defined to have a |LR|≥1 and a -log10 [p-value] > 2. a: CTRL siRNA vs. untreated CTRL revealed no off-target effects. The siRNAs A1 revealed 22 genes to be deregulated (b), the siRNA D1 - 8 genes (c), the siRNA D2 - 25 genes (d), the siRNA Q3 - 58 genes (e) & the siRNA Q4 - 42 genes (f). (1.20 MB PDF) [file pone.0014272.s003.pdf]

Figure S4

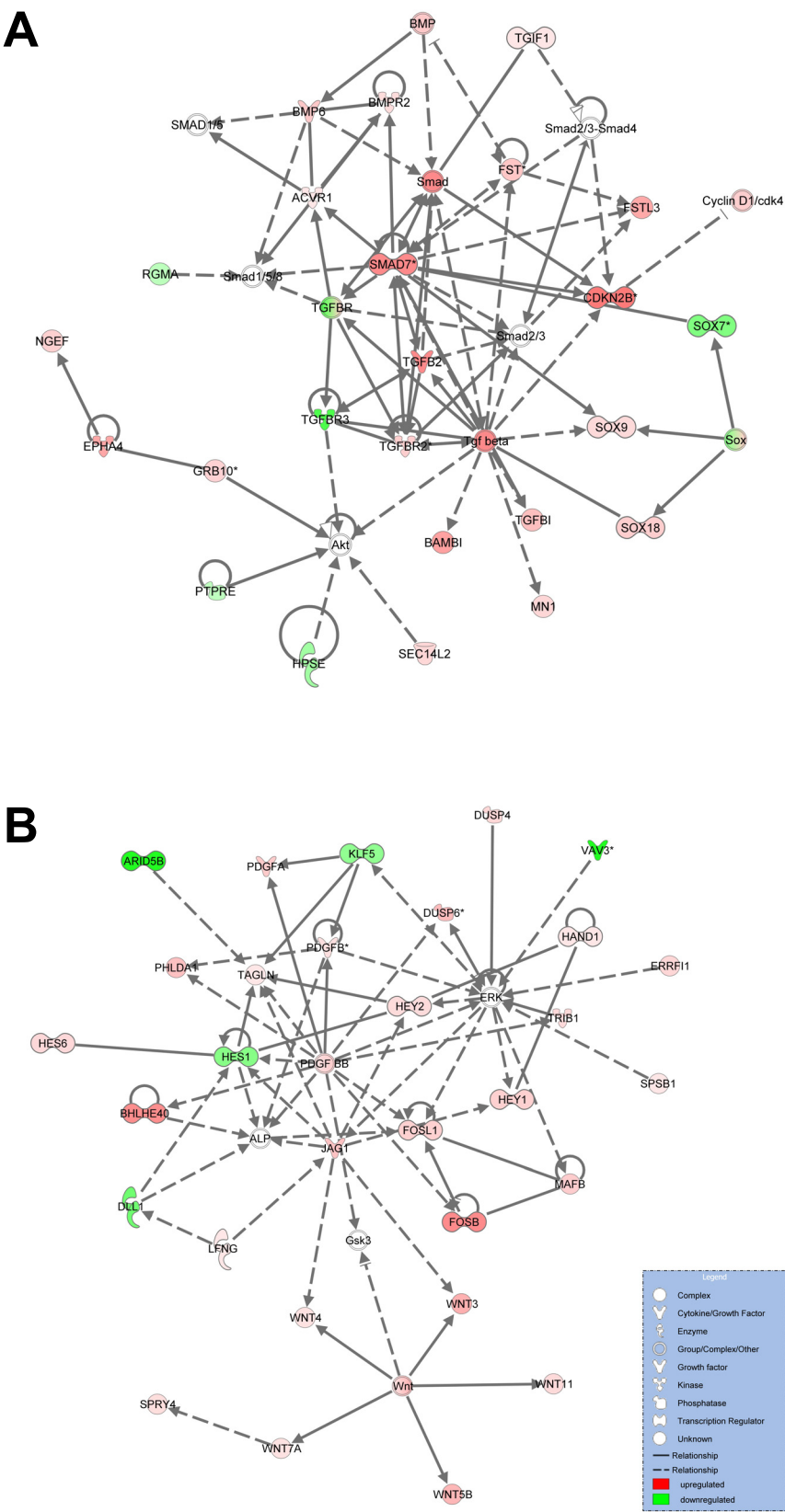

Supplement: Figure S4 — Ingenuity on-target Analysis. Networks of interacting and regulated molecules from the on-target signature as generated by Ingenuity Pathway Analysis. Molecules are represented as nodes and the biological relationship between two nodes is represented as an edge (line). All edges are supported by at least 1 literature reference. The intensity of the node color indicates the degree of up- (red) or down- (green) regulation. Nodes are displayed using various shapes that represent the functional class of the gene product. a: A network of molecules directly related to the canonical TGF- β signaling pathway containing genes involved in cell signaling, connective tissue development and function and in skeletal tissue development and function. b: A network of molecules of the WNT and the Erk/MAPK signaling pathways containing genes responsible for organ-, tissue and cellulare development. (0.48 MB PDF) [file pone.0014272.s004.pdf]

Figure S6

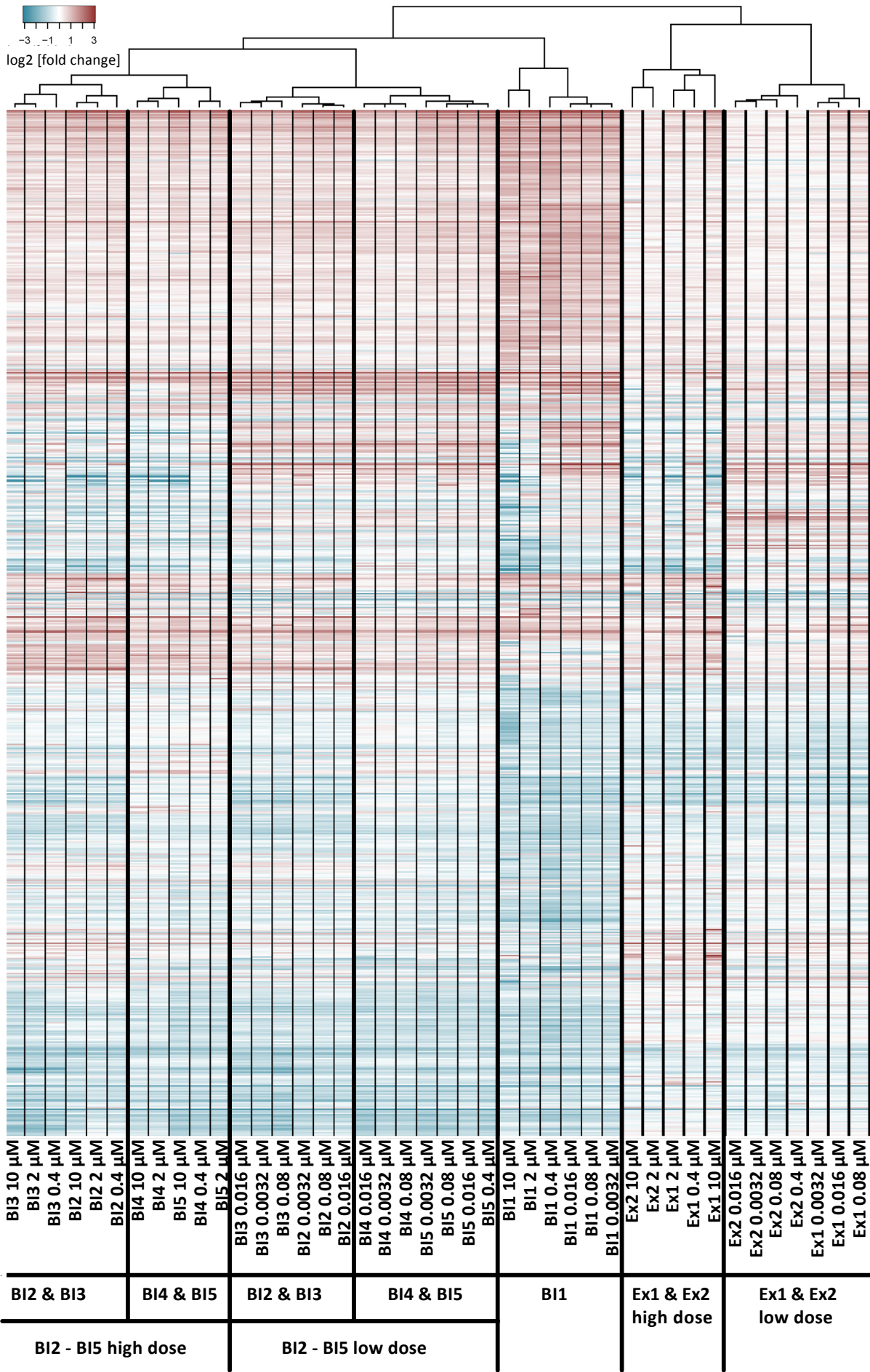

Supplement: Figure S6 — Hierarchical Clustering. Hierarchical clustering of 4314 significant deregulated genes (|LR| ≥ 1 & p-value < 0.01) after NCE treatment and TGF-β stimulation for 2h in HaCaT cells. The expression patterns of the different NCE treated cells reveal several intersections in gene regulation. The five indolinones (BI1-BI5) are grouped and separated from the pyridopyrimidinones (Ex1 & Ex2). Expression patterns are grouped in high vs. low dose fractions. The indolinone BI1 separates from the other class members, which can be further divided into two subgroups containing BI2 and BI3 and BI4 and BI5, respectively. Blue indicates decreased expression relative to untreated cells, red indicates increased expression. (1.50 MB PDF) [file pone.0014272.s006.pdf]

Figure S7

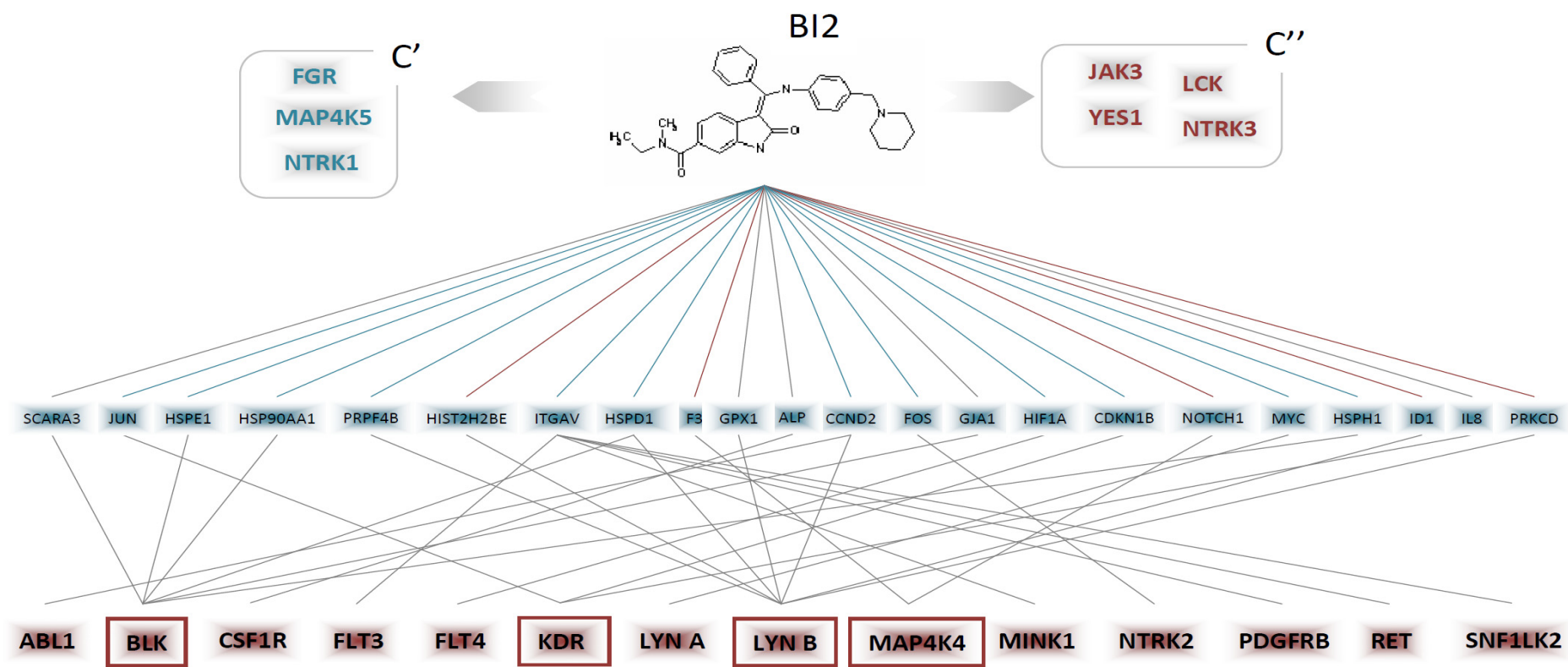

Supplement: Figure S7 — In silico prediction of kinase hits. Projections of BI2-specifically regulated kinase surrogate marker genes. Indicated are all 21 inhibited kinases: 3 kinases with no affection of known surrogate marker genes (c'), 4 kinases of which no surrogate markers are described (c') and 14 kinases with de-regulated surrogate markers genes (blue line = transcriptional down-regulation, red line = transcriptional up-regulation, red box = in silico predicted and biochemically confirmed BI2-specific kinase hits). (0.43 MB PDF) [file pone.0014272.s007.pdf]
