# Supplementary material for: Phenocopy – A Strategy to Qualify Chemical Compounds during Hit-to-Lead and/or Lead Optimization
Source: PLoS One. 2010 Dec 10;5(12):e14272. doi: 10.1371/journal.pone.0014272 (PMC3000806; doi:10.1371/journal.pone.0014272)

Figure S5

A TGF-β Effect (On Target Effect)

> No regulation by compounds only (*)
> Dose-dependent down-regulation of Serpine-1
> High dose titration reaches w/o TGF-β levels of Serpine-1 (#)

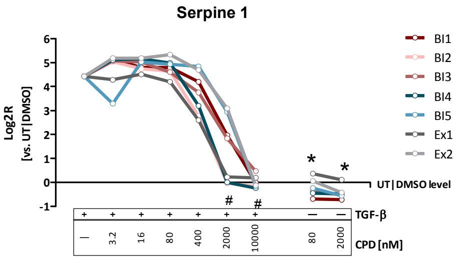

B Off Target Effect

> ILMN_1829845 is dose-dependent regulated by compound BI1 only (*)
> Regulation of ILMN_1829845 is not TGF-β-dependent (#)
> All other compound do not affect the expression of ILMN_1829845

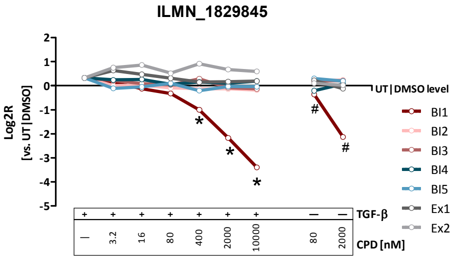

C On & Off Target Effect (additive)

> LAMC2 is regulated ~2-fold by TGF-β signaling (on-target) (*)
> All compounds show a dose-dependent down-regulation of LAMC2
> Compound BI1 down-regulates LAMC2 also TGF-β-independent (off-target)(#)
> For BI1 an additive effect of on- and off-target effect is observed ($)

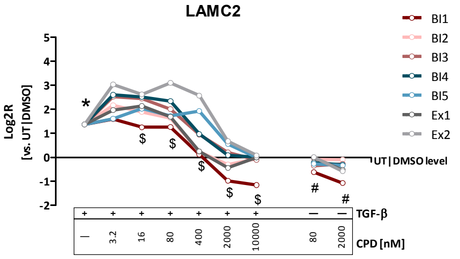

D On & Off Target Effect (inverse)

> BI1 shows a dose-dependent down-regulation of ARID5B in a TGF-β- independent manner (off-target) (*)
> TGF-β stimulation revealed a strong down-regulation of ARID5B (on target) (#)
> the regulation of ARID5B is not affected by BI1 titration - inverse effect (on target vs. off target effect) (&)
> all other compounds are able to inhibit the TGF-β effect (on target) dose-dependently ($)

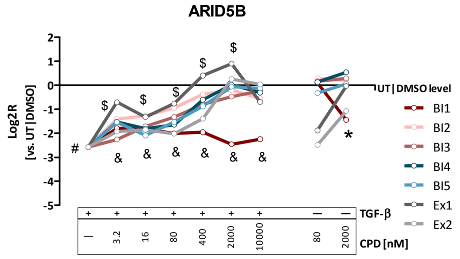

E On & Off Target Effect (bipolar)

> All compounds are inducing the expression of C9ORF152 in a TGF-β independent manner (off-target) (*)
> Under TGF-β stimulation the compounds BI1, BI2 &Ex1 tend to induce the C9ORF152 expression in a dose-dependent manner at lower concentration (on target) (#)
> BI1, BI2 & Ex1 show a bipolar regulation of C9ORF152: they induce the expression at lower level, whereas they down-regulate C9ORF152 at high doses ($)

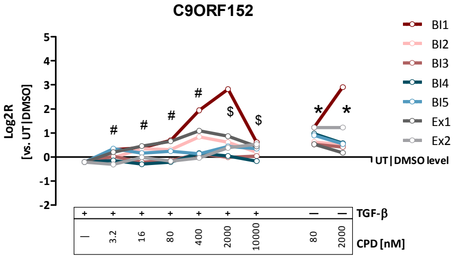

F Off Target Effect (common)

> All compounds are reducing SCARNA9 expression in TGF-β-independent manner (off-target) (*)
> TGF-β stimulation only showed no regulation of SCARNA9 expression (#)
> All compounds are down-regulating SCARNA9 in dose-independent manner ($): treatment effect?

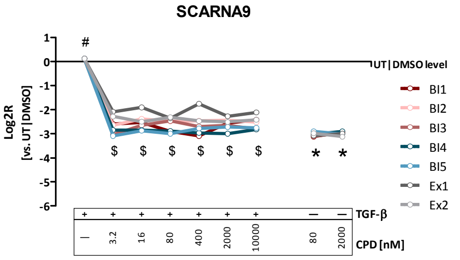

Supplement: Figure S5 — Case profile definition. NCE treatment and TGF-β stimulation resulted in six different cases profiles of gene regulation: representative examples for on-target effects triggered by TGF-β (a); Off-target effects triggered by a NCE (b); Integrated effects for on and off-targets in an additive (c), inverse (d) and bipolar (e) manner; common off-target effects induced by all 7 NCEs in a dose-independent manner (f). (0.12 MB PDF) [file pone.0014272.s005.pdf]
