# Supplementary material for: Phenocopy – A Strategy to Qualify Chemical Compounds during Hit-to-Lead and/or Lead Optimization
Source: PLoS One. 2010 Dec 10;5(12):e14272. doi: 10.1371/journal.pone.0014272 (PMC3000806; doi:10.1371/journal.pone.0014272)
Supplement: Table S3 — Each compound was profiled against a panel of 239 kinases. The kinases inhibited by each compound at 2μM and 200 nM are listed. (0.07 MB PDF) [file pone.0014272.s010.pdf]

Table S3: Each compound was profiled against a panel of 239 kinases. The kinases inhibited by each compound at 2µM and 200nM are listed.

NCE	Kinase	% Inhibtion [2 µM]	% Inhibtion [200nM]
BI1	ABL1	97	89
BI1	ALK	97	99
BI1	AMPK A1/B1/G1	98	92
BI1	AURKA (Aurora A)	98	87
BI1	AURKB (Aurora B)	101	80
BI1	AURKC (Aurora C)	93	86
BI1	AXL	98	88
BI1	BLK	98	92
BI1	BRAF	98	87
BI1	BRSK1 (SAD1)	93	76
BI1	CAMK2D (CaMKII delta)	100	81
BI1	CDK7/cyclin H/MNAT1	97	82
BI1	CHEK1 (CHK1)	94	92
BI1	CLK1	97	98
BI1	CLK2	98	99
BI1	FER	102	94
BI1	FGFR1	97	89
BI1	FGFR2	103	94
BI1	FGFR3	97	76
BI1	FGR	101	102
BI1	FLT1 (VEGFR1)	95	70
BI1	FLT3	100	101
BI1	FLT4 (VEGFR3)	102	104
BI1	FYN	97	90
BI1	GRK7	92	61
BI1	GSK3A (GSK3 alpha)	99	80
BI1	GSK3B (GSK3 beta)	99	93
BI1	HCK	100	80
BI1	JAK2	91	74
BI1	JAK3	101	97
BI1	KDR (VEGFR2)	100	98
BI1	LCK	103	98
BI1	LRRK2	98	88
BI1	LYN A	99	87
BI1	LYN B	99	91
BI1	MAP2K1 (MEK1)	101	93
BI1	MAP2K2 (MEK2)	100	98
BI1	MAP3K8 (COT)	95	85
BI1	MAP4K2 (GCK)	102	99
BI1	MAP4K4 (HGK)	104	102
BI1	MAP4K5 (KHS1)	101	105
BI1	MARK1 (MARK)	102	83
BI1	MARK2	92	63
BI1	MARK3	105	91
BI1	MARK4	103	95
BI1	MELK	93	99
BI1	MERTK (cMER)	102	98
BI1	MINK1	103	93
BI1	MUSK	90	75
BI1	NTRK1 (TRKA)	101	98
BI1	NTRK2 (TRKB)	102	98
BI1	NTRK3 (TRKC)	103	96
BI1	PDGFRA (PDGFR alpha)	97	87
BI1	PDGFRB (PDGFR beta)	98	87
BI1	PHKG1	91	72
BI1	PKN1 (PRK1)	98	86
BI1	PRKCA (PKC alpha)	96	67
BI1	PRKCN (PKD3)	95	104
BI1	PRKCQ (PKC theta)	100	73
BI1	PRKD1 (PKC mu)	102	99
BI1	PRKD2 (PKD2)	98	97
BI1	PRKG1	99	79
BI1	RAF1 (cRAF) Y340D Y341D	97	91
BI1	RET	102	102
BI1	RPS6KA1 (RSK1)	99	91
BI1	RPS6KA2 (RSK3)	100	97
BI1	RPS6KA3 (RSK2)	94	85
BI1	SNF1LK2	103	88
BI1	SRC	97	87
BI1	STK22D (TSSK1)	106	93
BI1	STK25 (YSK1)	104	99
BI1	STK3 (MST2)	100	98
BI1	STK4 (MST1)	103	102
BI1	TYK2	95	80
BI1	YES1	104	99
BI2	ABL1	96	67
BI2	BLK	93	57
BI2	CSF1R (FMS)	97	81
BI2	FGR	99	75
BI2	FLT3	96	70
BI2	FLT4 (VEGFR3)	100	90
BI2	JAK3	94	90
BI2	KDR (VEGFR2)	98	80
BI2	LCK	101	99
BI2	LYN A	96	58
BI2	LYN B	97	66
BI2	MAP4K4 (HGK)	102	78
BI2	MAP4K5 (KHS1)	100	104
BI2	MINK1	101	62
BI2	NTRK1 (TRKA)	100	86
BI2	NTRK2 (TRKB)	106	85
BI2	NTRK3 (TRKC)	103	99
BI2	PDGFRB (PDGFR beta)	96	58
BI2	RET	95	57
BI2	SNF1LK2	103	93
BI2	YES1	95	61
BI3	ABL1	93	67
BI3	BLK	97	85
BI3	CSF1R (FMS)	94	82
BI3	FGR	99	91
BI3	FLT3	93	66
BI3	FYN	93	74
BI3	LCK	100	100
BI3	LYN A	97	79
BI3	LYN B	97	78
BI3	MAP4K5 (KHS1)	93	77
BI3	MELK	93	80
BI3	NTRK3 (TRKC)	100	78
BI3	PDGFRA (PDGFR alpha)	99	91
BI3	PDGFRB (PDGFR beta)	94	76
BI3	RET	98	79
BI3	SNF1LK2	103	96
BI3	YES1	100	90
BI4	CSF1R (FMS)	95	87
BI4	FGR	93	80
BI4	FLT3	91	69
BI4	FLT4 (VEGFR3)	94	71
BI4	KDR (VEGFR2)	95	81
BI4	LCK	99	89
BI4	MAP4K4 (HGK)	99	59
BI4	MAP4K5 (KHS1)	99	93
BI4	NTRK1 (TRKA)	95	83
BI4	NTRK2 (TRKB)	97	89
BI4	NTRK3 (TRKC)	103	94
BI4	SNF1LK2	100	82
BI5	ABL1	90	75
BI5	CSF1R (FMS)	94	84
BI5	FGR	94	84
BI5	FLT3	96	90
BI5	FLT4 (VEGFR3)	93	74
BI5	KDR (VEGFR2)	95	81
BI5	LCK	98	90
BI5	MAP4K4 (HGK)	100	69
BI5	MAP4K5 (KHS1)	101	99
BI5	MINK1	94	60
BI5	NTRK1 (TRKA)	95	82
BI5	NTRK2 (TRKB)	98	88
BI5	NTRK3 (TRKC)	101	93
BI5	RET	100	93
BI5	SNF1LK2	100	83
Ex1	ABL1	94	100
Ex1	ABL2 (Arg)	99	105
Ex1	ACVR1B (ALK4)	94	90
Ex1	BLK	96	100
Ex1	BMX	99	97
Ex1	BRAF	102	100
Ex1	BTK	98	100
Ex1	CSF1R (FMS)	109	105
Ex1	EPHA1	91	99
Ex1	EPHA2	98	100
Ex1	EPHA5	98	101
Ex1	EPHA8	97	99
Ex1	EPHB1	95	101
Ex1	EPHB4	96	98
Ex1	FES (FPS)	102	86
Ex1	FGFR1	100	97
Ex1	FGFR2	101	99
Ex1	FGFR3	98	86
Ex1	FGFR4	92	79
Ex1	FGR	96	105
Ex1	FLT3	96	85
Ex1	FLT4 (VEGFR3)	100	94
Ex1	FRK (PTK5)	94	101
Ex1	FYN	98	93
Ex1	JAK2	98	94
Ex1	JAK3	92	83
Ex1	KDR (VEGFR2)	109	88
Ex1	LCK	101	96
Ex1	LYN A	100	101
Ex1	LYN B	90	102
Ex1	MAP2K1 (MEK1)	92	58
Ex1	MAP2K2 (MEK2)	98	68
Ex1	MAP3K9 (MLK1)	106	89
Ex1	MAP4K4 (HGK)	103	92
Ex1	MAP4K5 (KHS1)	103	100
Ex1	MAPK11 (p38 beta)	95	90
Ex1	MAPK14 (p38 alpha)	101	99
Ex1	MARK1 (MARK)	90	83
Ex1	MARK3	96	92
Ex1	MARK4	100	93
Ex1	MINK1	104	77
Ex1	MST4	97	62
Ex1	MYLK2 (skMLCK)	107	95
Ex1	NEK2	106	84
Ex1	PDGFRA (PDGFR alpha)	99	101
Ex1	PDGFRB (PDGFR beta)	100	91
Ex1	PRKCN (PKD3)	90	88
Ex1	PRKD1 (PKC mu)	96	92
Ex1	PRKD2 (PKD2)	95	88
Ex1	PTK6 (Brk)	94	107
Ex1	RAF1 (cRAF) Y340D Y341D	103	98
Ex1	RET	99	99
Ex1	SNF1LK2	100	104
Ex1	SRC	91	102
Ex1	SRMS (Srm)	94	90
Ex1	STK22D (TSSK1)	92	74
Ex1	STK24 (MST3)	98	58
Ex1	SYK	90	87
Ex1	TAOK2 (TAO1)	103	96
Ex1	YES1	96	102
Ex2	ABL1	91	100
Ex2	ABL2 (Arg)	94	101
Ex2	BRAF	95	74
Ex2	BTK	91	81
Ex2	CSF1R (FMS)	105	102
Ex2	EPHA2	94	95
Ex2	EPHA8	93	93
Ex2	FGFR2	94	61
Ex2	FGR	99	102
Ex2	FYN	97	84
Ex2	LCK	98	95
Ex2	LYN A	97	98
Ex2	MYLK2 (skMLCK)	102	65
Ex2	RAF1 (cRAF) Y340D Y341D	98	93
