# Supplementary material for: Phenocopy – A Strategy to Qualify Chemical Compounds during Hit-to-Lead and/or Lead Optimization
Source: PLoS One. 2010 Dec 10;5(12):e14272. doi: 10.1371/journal.pone.0014272 (PMC3000806; doi:10.1371/journal.pone.0014272)
Supplement: Methods S1 — (0.08 MB DOC) [file pone.0014272.s011.doc]

**Methods S1**

*qRT- PCR*

mRNA expression levels of TGF-βR1 and PAI-1 were determined by qRT-PCR analysis using a 7900HT Fast Real-Time PCR System (Applied Biosystems) and the Universal ProbeLibrary System (Roche). Gene specific forward- and reverse primer sequences were designed using the Universal Probe Library Assay Design Center (Roche). Total RNA was transcribed into cDNA using the High Capacity cDNA Reverse Transcription Kit (Applied Biosystems) according to the manufacture’s instructions. qRT-PCR is carried out in a final volume of 12 µl in three replicates for each cDNA sample. Levels of RNA polymerase II were used for normalization of the data. ΔΔCT method was used to relatively quantify mRNA levels of treated samples compared to untreated controls.

*ELISA analysis of PAI-1 and pSmad*

To analyze SMAD2/3 phosphorylation after TGF-β stimulation and/or NCE treatment cells were lysed in 100 µl Cell Lysis Buffer (Cell Signaling) supplemented with 1 mM PMSF (Sigma). 96-well plates (Nunc MaxiSorp™) were coated with anti-SMAD2/3 monoclonal antibody (1 µg/ml; BD Bioscience) for 24 h at 4 °C. To reduce unspecific binding the wells were blocked with PBS + 2% BSA for 2h at RT. After washing three times with PBS + 0.1% Tween20, the protein lysate was added and incubated for 2h at RT. Wells were washed three times with wash buffer and incubated with an anti-phoshpo-SMAD2/3 specific rabbit antisera (Eurogentec) diluted in PBS + 0.2% BSA + 0.02% Tween20 and incubated for 2h at RT. A AP-conjugated mAb mouse anti-rabbit IgG (Sigma) was added and incubated for 2h at RT. pNPP Liquid Substrate System (Sigma) was added and developed in the dark at 37°C for 2h before the absorbance was measured at 405nm in a Synergy HT plate reader (BioTek).

To analyze protein expression of PAI-1 supernatants of TGF-β stimulated and optionally NCE treated cells were collected 1, 2, 4, 12 and 24h after treatment and analyzed using the PAI-1 Antigen Kit (Haemochrom Diagnostica), according to the manufacturer’s protocol.

TGF-β signature

To define genes deregulated by TGF-β signaling, three sequential filtering steps were applied to the log_2_ transformed expression values of each time point separately. 1) The first filtering is based on the comparison of TGF-β stimulated cells against untreated cells by linear models^1^ (FDR corrected^2^ p-value < 0.01 and |log_2_ ratio| ≥ 0.5). 2) A linear model was applied to the dose groups of each compound to extract all probes which are significantly deregulated (FDR-corrected p-value < 0.01) by at least one concentration compared to the respective control (cells treated with TGF-β and DMSO but no compound). 3) To detect probes with a dose dependent deregulation the likelihood ratio test statistic for monotonicity (R package IsoGene^3^) was used. IsoGene performs an isotonic regression based on the replicates for each concentration resulting in regression values μ_1_, μ_2_, …, μ_6_ for each probe and each compound treatment. Only probes that are significantly regulated by at least one compound with |μ_1_-μ_6_| ≥ 1 and an FDR corrected p-value < 0.01 are included in the further analysis. For each time point the probes that passed all three filters are pooled to the final TGF-β signature.

Off-target signature

To detect transcripts that are deregulated due to off-target effects of the compounds unstimulated cells (wotgf class) as well as TGF-β stimulated cells (tgf class) were considered. For the wotgf class, we compared 0.08μM just as 2μM compound treated cells to untreated cells (d_11_ and d_12_, respectively), and 2μM to 0.08μM compound treated cells (δ_1_) for each time point using linear models^1^ (FDR-corrected^2^ p-value < 0.01 and |log_2_ ratio| ≥ 1). The same comparisons were made based on the tgf class (d_21_, d_22_ and δ_2_). Transcripts that are up/down regulated by either compound treatment (wotgf_up_ and wotgf_down_, respectively) or by TGF-β stimulation together with compound treatment (tgf_up_ and tgf_down_, respectively) were detected based on the described comparisons as follows:

A transcript belongs to the class wotgf_up_ if either δ_1_ is significantly up regulated (δ1_up) or if δ_1_ is not significantly down regulated (¬δ1_down) but d_11_, d_12_, and δ_1_ indicate an increasing course of expression intensity for higher compound concentrations. That is, if ¬δ1_down holds true, five different trends render upregulation: 1) d_11_ and d_12_ are both significantly upregulated; 2) d_11_ but not d_12_ is significantly downregulated and log_2_ratio(δ_1_) ≥ 1, thereby showing an increasing trend of expression for increasing compound concentrations; 3) d_11_ but not d_12_ is significantly upregulated and log_2_ratio(δ_1_) > -1, allowing for a small but not significant decreasing trend for increasing compound concentration; 4) d_12_ but not d_11_ is significantly downregulated and log_2_ratio(δ_1_) ≥ 1; 5) d_12_ but not d_11_ is significantly upregulated and log_2_ratio(δ_1_) > -1. On the one hand, as soon as one of d_11_ or d_12_ is significantly upregulated (cases 3 and 5), we allow for a small amount of noise by claiming log_2_ratio(δ_1_) > -1. On the other hand, as soon as one of d_11_ or d_12_ is significantly downregulated (cases 2 and 4), we are more strict by claiming log_2_ratio(δ_1_) ≥ 1 to call a transcript as being upregulated.

Stated in a more mathematical fashion, transcripts upregulated within the wotgf class are defined as follows:

$$\text{wotgf}_{\text{up}}=\left[\begin{matrix} \delta_{\text{1\_up}}\text{ }\vee\\ \text{[(}\neg\delta_{\text{1\_down}}\text{) }\wedge\text{ ((d}_{\text{11\_up}}\text{ }\wedge\text{ d}_{\text{12\_up}}\text{)} \\ \text{ }\vee\text{(d}_{\text{11\_down}}\text{ }\wedge\neg\text{d}_{\text{12\_down}}\text{ }\wedge\text{log}_{2}\text{ratio}\left(\delta_{1}\right)\geq\text{1)} \\ \text{ }\vee\text{(d}_{\text{11\_up}}\text{ }\wedge\neg\text{d}_{\text{12\_up}}\wedge\text{ log}_{2}\text{ratio}\left(\delta_{1}\right)\text{ }>\text{ -1)} \\ \text{ }\vee\text{(}\neg\text{d}_{\text{11\_down}}\wedge\text{d}_{\text{12\_down}}\wedge\text{ log}_{2}\text{ratio}\left(\delta_{1}\right)\geq\text{1)} \\ \text{ }\vee\text{(}\neg\text{d}_{\text{11\_up}}\wedge\text{d}_{\text{12\_up}}\text{ }\wedge\text{ log}_{2}\text{ratio}\left(\delta_{1}\right)>\text{ -1))]} \end{matrix}\right]$$

The mirrored method was used to detect wotgf_down_ and the analogous methods are used to detect tgf_up_ and tgf_down_ based on the cells stimulated with TGF-β1.

$$\text{wotgf}_{\text{down}}=\left[\begin{matrix} \delta_{\text{1\_down}}\text{ }\vee\text{ } \\ \text{[(}\neg\delta_{\text{1\_up}}\text{) }\wedge\text{ ((d}_{\text{11\_down}}\text{ }\wedge\text{ d}_{\text{12\_down}}\text{)} \\ \text{ }\vee\text{(d}_{\text{11\_down}}\text{ }\wedge\neg\text{d}_{\text{12\_down}}\text{ }\wedge\text{ log}_{2}\text{ratio}\left(\delta_{1}\right)<\text{1)} \\ \text{ }\vee\text{(d}_{\text{11\_up}}\text{ }\wedge\neg\text{d}_{\text{12\_up}}\wedge\text{ log}_{2}\text{ratio}\left(\delta_{1}\right)\text{ }\leq\text{ -1)} \\ \text{ }\vee\text{(}\neg\text{d}_{\text{11\_down}}\wedge\text{d}_{\text{12\_down}}\text{ }\wedge\text{ log}_{2}\text{ratio}\left(\delta_{1}\right)<\text{1)} \\ \text{ }\vee\text{(}\neg\text{d}_{\text{11\_up}}\wedge\text{d}_{\text{12\_up}}\text{ }\wedge\text{ log}_{2}\text{ratio}\left(\delta_{1}\right)\leq\text{ -1))]} \end{matrix}\right]$$

$$\text{tgf}_{\text{up}}=\left[\begin{matrix} \delta_{\text{2\_up}}\text{ }\vee\text{ } \\ \text{[(}\neg\delta_{\text{2\_down}}\text{) }\wedge\text{ ((d}_{\text{21\_up}}\text{ }\wedge\text{ d}_{\text{22\_up}}\text{)} \\ \text{ }\vee\text{(d}_{\text{21\_down}}\wedge\neg\text{d}_{\text{22\_down}}\text{ }\wedge\text{ log}_{2}\text{ratio}\left(\delta_{2}\right)\text{ }\geq\text{1)} \\ \text{ }\vee\text{(d}_{\text{21\_up}}\text{ }\wedge\neg\text{d}_{\text{22\_up}}\wedge\text{ log}_{2}\text{ratio}\left(\delta_{2}\right)\text{ }>\text{ -1)} \\ \text{ }\vee\text{(}\neg\text{d}_{\text{21\_down}}\text{ }\wedge\text{d}_{\text{22\_down}}\wedge\text{ log}_{2}\text{ratio}\left(\delta_{2}\right)\text{ }\geq\text{1)} \\ \text{ }\vee\text{(}\neg\text{d}_{\text{21\_up}}\text{ }\wedge\text{d}_{\text{22\_up}}\text{ }\wedge\text{ log}_{2}\text{ratio}\left(\delta_{2}\right)\text{ }>\text{ -1))]} \end{matrix}\right]$$

$$\text{tgf}_{\text{down}}=\left[\begin{matrix} \delta_{\text{2\_down}}\text{ | } \\ \text{[(}\neg\delta_{\text{2\_up}}\text{) }\wedge\text{ ((d}_{\text{21\_down}}\text{ }\wedge\text{ d}_{\text{22\_down}}\text{)} \\ \text{ }\vee\text{(d}_{\text{21\_down}}\text{ }\wedge\neg\text{d}_{\text{22\_down}}\wedge\text{ log}_{2}\text{ratio}\left(\delta_{2}\right)<\text{1)} \\ \text{ }\vee\text{(d}_{\text{21\_up}}\wedge\neg\text{ d}_{\text{22\_up}}\wedge\text{ log}_{2}\text{ratio}\left(\delta_{2}\right)\text{ }\leq\text{ -1)} \\ \text{ }\vee\text{(}\neg\text{d}_{\text{21\_down}}\wedge\text{d}_{\text{22\_down}}\text{ }\wedge\text{ log}_{2}\text{ratio}\left(\delta_{2}\right)\text{ }<\text{1)} \\ \text{ }\vee\text{(}\neg\text{d}_{\text{21\_up}}\wedge\text{d}_{\text{22\_up}}\text{ }\wedge\text{ log}_{2}\text{ratio}\left(\delta_{2}\right)\text{ }\leq\text{ -1))]} \end{matrix}\right]$$

Based on transcripts for which

$$\text{(tgf}_{\text{up}}\text{ }\wedge\text{ wotgf}_{\text{up}}\text{)}\vee\text{ (tgf}_{\text{down}}\text{ }\wedge\text{ wotgf}_{\text{down}})\vee(\text{tgf}_{\text{up}}\text{ }\wedge\text{ wotgf}_{\text{down}})\vee(\text{tgf}_{\text{down}}\text{ }\wedge\text{ wotgf}_{\text{up}})$$

we defined the final off-target signatures. The profiles of the respective transcripts can be assigned to different categories as described in Supplementary Figure 5:

$$\begin{matrix} \text{tgf}_{\text{up}}\text{ }\wedge\text{ wotgf}_{\text{up}}\text{:additive or bipolar on- and off-target effect or (common) off-target effect,} \\ \text{tgf}_{\text{down}}\text{ }\wedge\text{ wotgf}_{\text{down}}:\text{additive or bipolar on- and off-target effect or (common) off-target effect,} \\ \text{tgf}_{\text{up}}\text{ }\wedge\text{ wotgf}_{\text{down}}:\text{ inverse or bipolar on- and off-target effect, and} \\ \text{tgf}_{\text{down}}\text{ }\wedge\text{ wotgf}_{\text{up}}:\text{inverse or bipolar on- and off-target effect.} \end{matrix}$$

Reference List

1. Smyth,G.K. Linear models and empirical bayes methods for assessing differential expression in microarray experiments. *Stat Appl Genet Mol Biol* **3**, (2004).

2. Benjamini,Y. & Hochberg,Y. Controlling the false discovery rate: A practiacal and powerful approach to multiple testing. *J. Roy. Statist. Soc. Ser. B* **57**, 289-300 (1995).

3. Lin,D. *et al.* Testing for trends in dose-response microarray experiments: a comparison of several testing procedures, multiplicity and resampling-based inference. *Stat Appl Genet Mol Biol* **6**, Article26 (2007).
